# Identification of New Polyacetylenes from *Dendropanax morbifera* with PPAR-α Activity Study

**DOI:** 10.3390/molecules29245942

**Published:** 2024-12-16

**Authors:** Donglan Piao, Isoo Youn, Thanh-Hau Huynh, Hyun Woo Kim, Sang Gyun Noh, Hae Young Chung, Dong-Chan Oh, Eun Kyoung Seo

**Affiliations:** 1 Graduate School of Pharmaceutical Sciences, College of Pharmacy, Ewha Womans University, Seoul 03760, Republic of Korea; parkdl@ewhain.net (D.P.); iyoun@ewha.ac.kr (I.Y.); 2 Natural Products Research Institute, College of Pharmacy, Seoul National University, Seoul 08826, Republic of Korea; 2019-22632@snu.ac.kr (T.-H.H.); dongchanoh@snu.ac.kr (D.-C.O.); 3 Department of Pharmacy and Research Institute for Drug Development, College of Pharmacy, Pusan National University, Busan 46241, Republic of Korea; khw124124@naver.com (H.W.K.); rskrsk92@pusan.ac.kr (S.G.N.); hyjung@pusan.ac.kr (H.Y.C.)

**Keywords:** *Dendropanax morbifera* Leveille, polyacetylene, AMP-activated protein kinase, cathepsin S, Peroxisome proliferator-activated receptor

## Abstract

*Dendropanax morbifera* Leveille is a traditional medicine used to treat migraine headache and dysmenorrhea. In this study, three polyacetylenes, methyl (10*E*,9*R*,16*R*)-16-acetoxy-9-hydroxyoctadeca-10,17-dien-12,14-diynoate (**1**), methyl (10*E*,9*R*,16*S*)-9,16-dihydroxyoctadeca-10-en-12,14-diynoate (**2**), and methyl (10*Z*,9*R*,16*S*)-9,16-dihydroxyoctadeca-10,17-dien-12,14-diynoate (**3**), were isolated from the aerial parts of *D*. *morbifera*, together with seven known compounds (**4**–**10**). Importantly, the isolates (**6** and **8**) were found in the family Araliaceae for the first time in this study. Compounds **1**−**10** were evaluated for their binding affinity to AMPK and CTSS receptors using in silico docking simulations. Only compound **7** increased the protein expression levels of PPAR-α, Sirt1, and AMPK when administered to HepG2 cells as a PPAR-α agonist. On the other hand, **7** did not produce any significant reduction in CTSS activity. This study could pave the way for the discovery of novel treatments from *D. morbifera* targeting PPAR-α and AMPK.

## 1. Introduction

*Dendropanax morbifera* Leveille belongs to the family Araliaceae and is an endemic species in Korea [1]. *D. morbifera* is an evergreen tree with a golden color, medium size (height of about 15 m), and leaves resembling duck palms [2]. The name of this plant, which is commonly known as Hwangchil in Korea, originates from Hwang meaning golden yellow and chil meaning coatings because it has been used as a natural golden coating. *D. morbifera* is a traditional medicine commonly used to treat migraine headache, dysmenorrhea, and rheumatoid arthritis [3]. In addition, *D. morbifera* has been reported to exhibit wide biological activities, such as anti-diabetic [4], anti-cardiac hypertrophy [5], cytotoxic [6], and anti-inflammatory properties [7].

More than 90 compounds have been identified in *D. morbifera*, including phenylpropanoids, flavonoids, and polyacetylenes [8]. Phenylpropanoids are secondary metabolites found in various plants, primarily synthesized through the shikimate pathway [9]. Enzymes such as oxygenases, ligases, and oxido-reductases ultimately generate a diverse array of phenylpropanoid compounds that exhibit cytotoxic [10], anti-oxidant [11], and anti-inflammatory activities [12]. Flavonoids are synthesized via two pathways: the shikimate pathway to produce the phenylpropanoid skeleton and the acetate pathway to provide 2-carbon unit building blocks [13]. They exhibit activities beneficial for treating cytotoxic conditions [14], cardiovascular disease [15], and metabolic diseases [16]. Polyacetylenes are typically unstable compounds with a unique carbon–carbon triple bond structure. Due to their extensive biological activities and novel biosynthetic pathways, these compounds have attracted considerable attention [17,18]. Isotope tracing studies indicated that most polyacetylenes originate from precursors of fatty acids and polyketides [17]. This class of the compounds has demonstrated efficacy in treating diabetes [19] and inflammation [20].

As nutritional supports and life expectancy grow, metabolic syndromes, such as T2 diabetes mellitus, hyperlipidemia, and cardiac diseases, are becoming more prevalent and now occur in nearly 25% of the adult population worldwide [21]. Although diet and exercise are the primary treatments for those diseases, pharmaceutical therapy is necessary to relieve severe obesity, dyslipidemia, diabetes, and hypertension. Current treatments, including glucagon-like peptide-1 agonists, insulin sensitizers, sodium glucose transporter-2 inhibitors, antiplatelet agents, HMG-CoA reductase inhibitors, and renin-angiotensin system blockers, are administered to control those diseases in patients. However, long-term use of those drugs and severe side effects burden the patients who use them [22].

Peroxisome proliferator-activated receptor (PPAR) is a transcription factor that controls various physiological processes in the body [23]. Among the three PPAR isoforms (α, β/δ, and γ), PPAR-α is predominantly expressed in the liver, heart, and kidney and is required for fatty acid/lipoprotein metabolism and peroxisomal proliferation [24]. AMP-activated protein kinase (AMPK) is an enzyme sensor that maintains cellular energy balance [25]. Several studies have reported that PPAR-α activators stimulate the AMPK signaling pathway, thereby regulating hepatic lipogenesis, adipocyte differentiation, and glucose uptake in muscle [25,26,27]. Therefore, PPAR-α agonists can stimulate AMPK in ways favorable to the prevention of metabolic syndrome. On the other hand, cathepsin S (CTSS) belongs to the peptidase C1 family of cysteine cathepsins, which are lysosomal cysteine proteases that function in the degradation and processing of proteins [28]. CTSS is predominantly found in antigen-presenting cells such as macrophages and T cells [29], and it is stable at a neutral pH. CTSS has been found to correlate with diabetes and adipogenesis [30,31].

As mentioned above, researchers have reported that *D*. *morbifera* is effective against various metabolic syndromes, and AMPK and CTSS levels correlate with those diseases [32,33,34,35,36]. This study aims to find active ingredients from *D*. *morbifera* and elucidate the relationships between those compounds and AMPK, PPAR-α, and CTSS. Therefore, compounds were isolated, and then their structures were elucidated using NMR, HR-MS, and ECD techniques. The biological activity of the *D. morbifera*–derived compounds was investigated by examining their effects on AMPK, PPAR-α, and CTSS in computer simulation and cell-based analyses.

## 2. Results and Discussion

**Figure 1:**
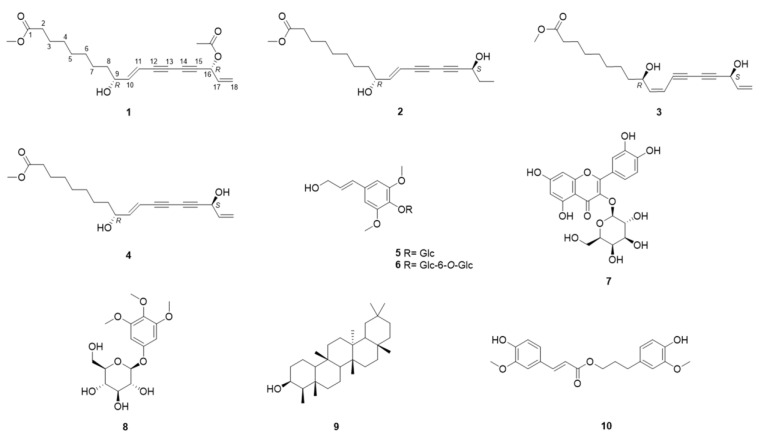
The structures of compounds **1**–**10**.

In this study, three new polyacetylenes (**1**–**3**) were isolated from the aerial parts of *D*. *morbifera*, together with seven known compounds (**4**–**10**) (Figure 1). The known compounds were identified as methyl (10*E*,9*R*,16*S*)-9,16-dihydroxyoctadeca-10,17-dien-12,14-diynoate (**4**) [37], syringin (**5**) [38], syringinoside (**6**) [38], hyperoside (**7**) [39], koaburside (**8**) [40], epifriedelanol (**9**) [41], and dihydroconiferyl ferulate (**10**) [42]. Notably, **6** and **8** were reported for the first time in the family Araliaceae. According to previous research, **4** significantly inhibits melanin production [37]. Compound **5** possesses anti-inflammatory [43], cytotoxic [44], and immunomodulatory properties [45]. Compound **6** was found in *Wikstroemia sikokiana* in 1988 [38], and it has also been isolated from other plants, such as *Stelleropsis iranica* and *Daphne oleoides* [46,47]. Compound **7** has been studied extensively for its various biological activities, including anti-inflammatory [48], antidepressant-like [49], and antibacterial effects [50]. Compound **8** has been isolated from *Betula platyphylla* and *Walsura robusta* [51,52] and shown antioxidant activity [52]. Compound **9** has been reported to have anti-aging effects [53], as well as antibacterial and anti-tumor properties [54,55]. Compound **10** can be used as a cancer stem cell inhibitor for the treatment of breast cancer [6].

### 2.1. Structure Elucidation

**Figure 2:**
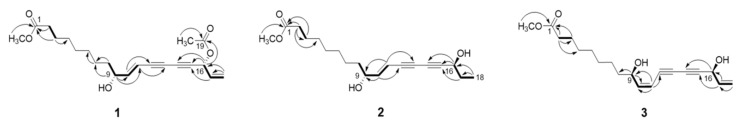
Key ^1^H−^1^H COSY (in bold) and HMBC (black arrows) correlations in **1**–**3**.

**Figure 3:**
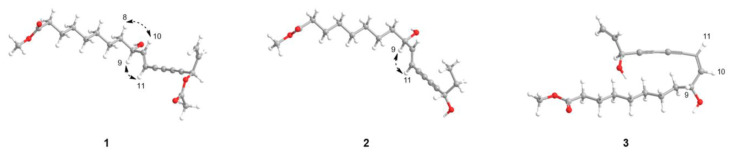
Key ^1^H−^1^H NOESY correlations in **1**–**3**.

Compound **1** was obtained as a brown amorphous powder with a molecular ion peak at *m*/*z* 383.1835 [M + Na]^+^ in the HRESIMS, showing its molecular formula as C_21_H_28_NaO_5_ with 8 degrees of unsaturation. In the ^1^H spectrum of **1** (Table 1), two sets of olefinic proton signals appeared at *δ*_H_ 6.34 (1H, dd, *J* = 15.9, 5.7 Hz), 5.88 (1H, ddd, *J* = 16.9, 13.0, 5.7 Hz), 5.76 (1H, ddd, *J* = 15.9, 1.6, 0.8 Hz), 5.55 (1H, dt, *J* = 16.9, 1.1 Hz), and 5.35 (1H, dt, *J* = 10.3, 1.1 Hz) [37]. Two oxygenated methines were observed at *δ*_H_ 4.20 (1H, m)/*δ_C_* 72.0 (C-9) and 5.96 (1H, dd, *J* = 5.8, 1.0 Hz)/64.7 (C-16), together with seven methylene groups at *δ*_H_ 2.30 (2H, t, *J* = 7.5 Hz,)/*δ*_C_ 34.1 (C-2), 1.62 (2H, m)/24.9 (C-3), 1.31 (2H, m)/29.1 (C-4), 1.31 (2H, m)/29.0 (C-5), 1.25 (2H, s)/29.2 (C-6), 1.31 (2H, m)/25.1 (C-7), and 1.55 (2H, d, *J* = 7.2 Hz)/36.8 (C-8). The COSY correlations of **1** built the connectivity between H-2/H-3, H-8/H-9, H-9/H-10, H-10/H-11, H-16/H-17, and H-17/H-18 (Figure 2). In the HMBC spectrum of **1**, correlations between H-2/C-1 and H-3/C-1 established the position of a carboxyl group next to C-2, and another cross-peak from a methoxy group (*δ*_H_ 3.67) to C-1 (*δ*_C_ 174.3) suggested the attachment of a methoxy group at C-1 (Figure 2). Furthermore, HMBC correlations between H-10/C-12, H-11/C-13, H-16/C-14, H-16/C-15, and H-17/C-15 indicated the existence of two consecutive acetylene functionalities (C-12 to C-15) [56]. The positions of double bonds were confirmed by the HMBC correlations from H-9 to C-10 and C-11, H-18 to C-16 and C-17, and H-17 to C-15. The Δ^10,11^ double bond was assigned as an *E* configuration by the large coupling constant (*J*_H-10/H-11_ = 15.9 Hz). It was also supported by NOE correlations between H-8/H-10 and H-9/H-11, suggesting that the olefinic protons were in the *E* configuration (Figure 3). Careful comparison of the 1D and 2D NMR spectra of **1** with those of **4** revealed similarities as a polyacetylene [37]. A major difference between **1** and **4** was the presence of an acetoxy group (*δ*_H_ 2.11 and *δ*_C_ 169.5 and 20.9) in the former. The location of the acetoxy group was assigned to C-16, as evidenced by the HMBC correlations from H-16 (*δ*_H_ 5.96) to 16-OCOCH_3_ (*δ*_C_ 169.5) and 16-OCOCH_3_ (*δ*_H_ 2.11) to 16-OCOCH_3_. 

The HRESIMS exhibited that the molecular formula of **2** is C_19_H_30_O_5_ based on the molecular ion peak at *m*/*z* 338.3419 [M + H_2_O]^+^. The ^1^H NMR and ^13^C NMR spectra of **2** (Table 1) were similar to those of **4**. Unlike **4**, **2** lacked signals for an olefinic group, with methylene (*δ*_H_ 1.58/*δ*_C_ 30.3, C-17) and methyl (*δ*_H_ 0.90/*δ*_C_ 9.4, C-18) groups observed in **2** instead. The presence of an ethyl group attached to the C-16 position was also established by COSY correlations between H-16/H-17 and H-17/H-18 and HMBC correlations from H-18 to C-16 and H-18 to C-17 (Figure 2). The NOE correlation observed between H-9 and H-11 suggested an *E* configuration of the olefinic protons (Figure 3).

Compound **3** was obtained as a brown amorphous solid, with a molecular ion peak at *m*/*z* 336.2172 [M + H_2_O]^+^ from the HRESIMS in agreement with the molecular formula C_19_H_28_O_5_. Although thorough 1D and 2D NMR interpretation of **3** showed a planar structure similar to **4**, the coupling constant between H-10 (*δ*_H_ 6.10) and H-11 (5.66) was 11.1 Hz, indicating the double bond as a *Z* geometric configuration [56]. There was no clear NOE correlation between H-8/H-10 and H-9/H-11, suggesting that the olefinic group was in the *Z* configuration (Figure 3).

A previous study reported the use of Mosher’s derivatization method and ECD calculations to determine the absolute stereochemistry of polyacetylene compounds [56]. Based on that study, the absolute stereochemistry of compounds **1**–**4** was determined. By comparing the ECD spectra with the calculated values, two possible configurations, “9*R*16*R*” and “9*R*16*S*”, were identified. Because the ECD spectra had limitations in definitively determining the absolute configuration between those two possibilities, a DP4+ simulation was used for the final structural assignment. A DP4+ simulation compares the calculated NMR chemical shifts of candidate structures with experimental data to determine which structure matches the experimental data and thereby assign the structural configuration [57]. DP4+ simulations provide advantages by preventing incorrect assignments and offering additional support for newly assigned structures, particularly when experimental data are uncertain [58].

**Figure 4:**
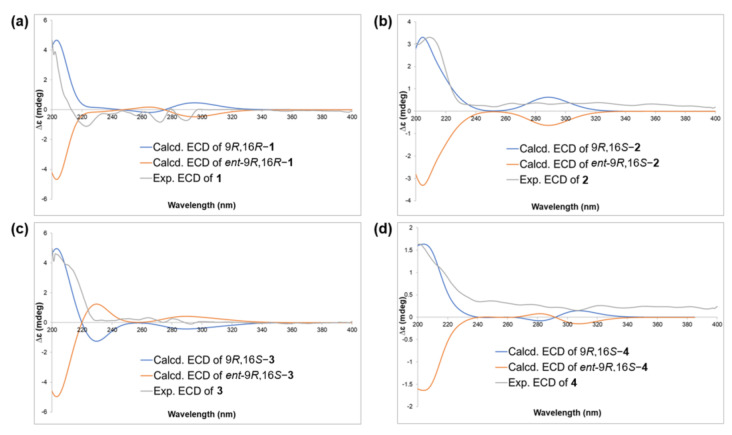
The ECD calculations of (**a**) **1**, (**b**) **2**, (**c**) **3**, and (**d**) **4**.

We calculated the two possible diastereomers (9*R*16*S* and 9*R*16*R*) for each structure by using a conformational search and geometric optimization to determine their relative configurations. The diastereomer with the highest probability was then subjected to further ECD calculations to elucidate the absolute configuration. The ECD spectra of the enantiomers were automatically generated without additional calculations. The DP4+ prediction results indicate that **2**–**4** share the same relative configuration (9*R*16*S*) with high confidence (Tables S4–S12 and Figures S30–S35). Compound **1**, on the other hand, exhibited the opposite relative configuration (9*R*16*R*) with a high probability (99%) (Tables S1–S3 and Figures S28 and S29). The ECD calculations confirmed the absolute configurations of **2**–**4** as 9*R*16*S* and that of **1** as 9*R*16*R* (Figure 4).

The polyacetylene compounds from *D. morbifera* are composed of 17 or 18 carbons, each containing two alkyne and two alkene functional groups, including (3*S*)-falcarinol, (3*S*,8*S*)-falcarindiol, and (3*S*)-diynene [59], most of which usually exist in a *cis* form [59,60,61,62]. In this study, the alkene functional group at C-10 and C-11 are in the *trans* form, except in **3**. Although **4** has been isolated before, its absolute configuration was not determined [37]. Therefore, we analyzed the absolute configuration of **4** using ECD calculations and DP4+ simulations. Polyacetylenes have been discovered in various plants, including *Bupleurum chinense* [63], *Carthamus tinctorius* [64], and *Codonopsis pilosula* [65]. Polyacetylenes isolated from natural products exhibit a range of biological properties, such as HIV reverse transcriptase inhibition and antibacterial activity [17,18,19,20]. Notably, C_17_- and C_18_-polyacetylenes have been reported to possess potential cytotoxic properties [17,20,66]. It has also been reported that polyacetylenes act as phytoalexins, protecting plants from diseases and external factors [67]. Additionally, in the ecological environment, they play a role in preventing barnacle larvae by causing biofouling [68].

### 2.2. In Silico Molecular Docking Results

Molecular docking can be used to identify potential drugs for treating various diseases. To explore the potential effects of *D*. *morbifera* on metabolic syndromes, in silico docking simulations were conducted using compounds **1**–**10**. These simulations aimed to examine the interactions and behavior of the compounds in AMPK and CTSS active sites. A control and compounds **1**–**10** were constructed in a 3D model, and molecular docking studies were conducted using four software programs (AutoDock Vina 1.1.2, AutoDock 4.2, LeDock 1.0, and Dock 6.12).

**Figure 5:**
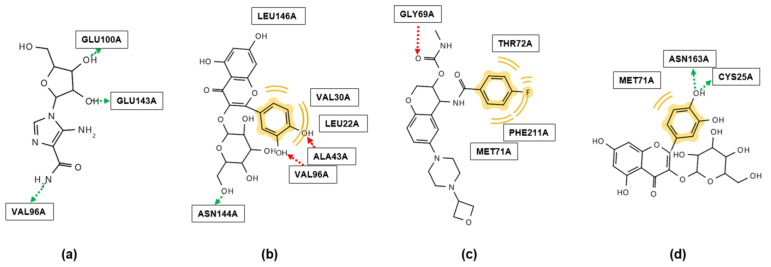
Binding interactions of (**a**) 5-amino-4-imidazolecarboxamide ribonucleoside (AICAR) and (**b**) **7** with AMP-activated protein kinase (AMPK) in in silico docking simulations in AutoDock 4.2. The binding sites of (**c**) LY3000328 and (**d**) **7** with cathepsin S (CTSS) in docking simulations in AutoDock 4.2. The green arrow indicates the hydrogen bond (H-bond) donor, the red arrow indicates the H-bond acceptor, and the yellow indicates hydrophobic interaction or Van der Waals force. (ALA, Alanine; ASN, Asparagine; GLU, Glutamic acid; LEU, Leucine; VAL, Valine; ASN, Asparagine; CYS, Cysteine; GLY, Glycine; MET, Methionine; PHE, Phenylalanine; THR, Threonine).

Among the compounds, **7** (−11.4 Kcal/mol) exhibited the highest affinity with AMPK, as shown in Table 2. Figure 5a,b show the molecular interaction models of 5-amino-4-imidazolecarboxamide ribonucleoside (AICAR, a well-known AMPK activator) and **7** with AMPK [69]. Although the control (AICAR) and **7** exhibit the same number of hydrogen bonds, **7** has a higher number of hydrophobic interactions, thereby demonstrating higher binding affinity. It is worth noting that both AICAR and **7** form the same hydrogen bond with the VAL96 residue, demonstrating that they are likely to have similar binding modes and thus related biological activities. This is the first study to conduct in silico simulations on compounds **1**–**10** with AMPK.

Table 3 clearly shows that each compound exhibited various levels of binding affinity against CTSS. Especially in the case of compound **7**, it is evident that its affinity for the binding sites is more stable than that of the control and other compounds. Figure 5c,d exhibit the molecular interactions of LY3000328 (Z-FL-COCHO) and **7** against CTSS. The control provided hydrophobic/Van der Waals interactions and acted as a hydrogen bond acceptor against CTSS (Figure 5c). As shown in Figure 5d, the B ring in **7** can form hydrophobic interactions/Van der Waals forces, as well as hydrogen bonds with the CTSS receptor. To the best of our knowledge, this is the first study to conduct in silico simulations of compounds **1**–**10** against CTSS.

### 2.3. In Vitro Assay

**Figure 6:**
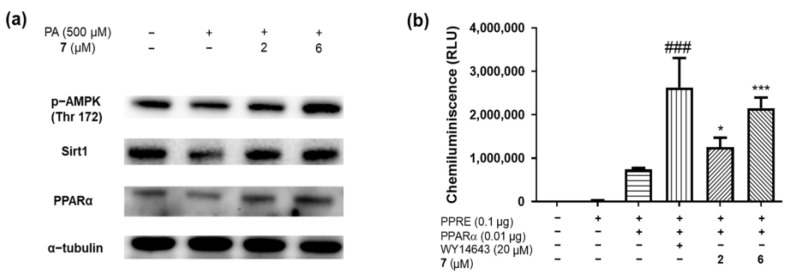
(**a**) The protein levels of AMPK, PPAR-α, and Sirt1 were measured in the presence of **7** using western blotting. (**b**) PPAR-α luciferase assay with 7. Data are represented as mean ± SEM. The minus (−) and plus (+) symbols indicate whether the factor is run at a low or high level, respectively. ### p < 0.001 versus PPRE with pcDNA treated cells; * p < 0.05 and *** p < 0.001 versus PPRE with PPARα DNA treated cells.

To evaluate the results from the in silico simulations, in vitro AMPK assays were performed. Compound **7**, which showed the highest binding affinity with AMPK in the in silico simulation, increased the protein levels of AMPK, PPAR-α, and Sirt1 as a PPAR-α activator, as shown by a PPAR-α luciferase assay (Figure 6a,b). Although a water extract and **7** were tested to investigate their inhibitory activity against CTSS, no positive result was observed. As PPAR-α activators can mitigate hepatic lipogenesis, adipose differentiation, and glucose metabolism in muscle by stimulating the AMPK signaling pathway [25,26,27], compound **7** can be a good candidate for structure-activity study and in vitro/in vivo experiments to find a cure for metabolic diseases.

Previous studies have demonstrated that a *D. morbifera* extract can activate AMPK and has therapeutic effects on diabetes, obesity, and nephrotoxicity [4,70,71]. Additionally, a patent suggests that it can enhance PPAR-α to regulate glucose activity [72]. Several studies have reported the biological effects of compounds **5** and **7** against AMPK. By regulating the AMPK pathway, **5** has shown potential to treat cardiac hypertrophy [73], diabetes [74], and obesity [75]. Compound **7** can be applied to hypoxia-related diseases [76], particulate matter–induced lung injury [77], kidney function damage [78], and enhanced insulin resistance and lipid synthesis through the AMPK signaling pathway [79].

Although the in vitro CTSS-inhibition screening of **7** did not show positive effects, the attempt itself has important research value because it was the first attempt to evaluate the in vitro effects of **7** on CTSS.

## 3. Materials and Methods

### 3.1. Plant Materials

The leaves and stems of *Dendropanax morbifera* were collected at Tree Travel Botanical Garden in Hadong-gun, Gyeongsangnam-do, Republic of Korea, in June 2021. A voucher specimen (No. EA391) was deposited at the Natural Product Chemistry Laboratory, College of Pharmacy, Ewha Womans University, Seoul, Republic of Korea.

### 3.2. General Experimental Procedures

Optical rotation was performed on a P-1010 polarimeter (Jasco, Tokyo, Japan), and the UV spectrum was recorded on a U-3000 spectrophotometer (Hitachi, Tokyo, Japan). NMR spectra were acquired on a Varian Unity Inova 400 MHz FT-NMR instrument (Agilent Technologies, Santa Clara, CA, USA) with TMS as an internal standard, and the data were processed in MestReNova 9.0 (Mestrelab Research SL, Santiago de Compostela, Spain). HRESIMS was performed on an Agilent 6230 Accurate-Mass TOF LC/MS system (Agilent). CD measurements were performed using an Applied Photophysics Chirascan-Plus CD spectrometer (Applied Photophysics, Surrey, UK). For column chromatography, Diaion HP-20 and Kieselgel 60 F254 (silica gel, 0.25 mm layer thickness) were purchased from Mitsubishi Chemical Co. (Tokyo, Japan) and Merck & Co. (Rahway, NJ, USA), respectively. MPLC was performed using CombiFlash (Teledyne Isco Inc., Lincoln, NE, USA) equipped with a RediSep Rf C18 column (130 g, Teledyne Isco Inc.) and a RediSep Rf normal phase silica column (24 g and 40 g). Preparative HPLC purification was conducted using a Waters 600 pump and a Waters 996 photodiode array detector (Waters, Milford, MA, USA) equipped with a YMC-Pack Pro C18 column and a YMC Actus pro C18 column (5 µm, 250 mm × 20 mm i.d., YMC Co., Kyoto, Japan).

### 3.3. Extraction and Isolation

The leaves and stems of *D. morbifera* (10 kg) were extracted with MeOH (3 × 55 L) at room temperature for 2 days. The solvent was subjected to rotary evaporation to obtain a MeOH extract (1.6 kg). The extract was suspended in 2 L of distilled water and fractionated with hexanes (11 × 2 L) and EtOAc (8 × 2 L), yielding a hexane extract (207.5 g), EtOAc extract (126.7 g), and aqueous extract (2.5 L).

The EtOAc extract (126 g) was subjected to silica gel column chromatography (CH_2_Cl_2_-MeOH, 1:0 to 0:1) to provide 15 fractions (A01–A15) and **7** (38 mg). Fractions A04 to A07 (25 g) were subjected to normal-phase column chromatography (hexane-EtOAc 0:1 to 1:0) to obtain 23 subfractions (A0401–A0423). Compound **9** (11 mg) was precipitated from subfraction A0402. Subfraction A0403 (162 mg) was separated by MPLC (hexane-EtOAc 1:0 to 0:1, 5 mL/min) to yield 14 subfractions (A040301–A040314). Subfraction A040302 (50 mg) was separated using silica gel (hexane-CH_2_Cl_2_ 1:0 to 0:1) and applied to preparative HPLC (YMC-Park PRO C18, MeOH-water, 75:25, 3 mL/min) to acquire **1** (1 mg, t*_R_* 85 min). Subfraction A0408 (3.4 g) was subjected to MPLC (hexane-acetone, 1:0 to 0:1, 5 mL/min) to yield 15 subfractions (A040801–A040815). Subfraction A040804 (1.3 g) was chromatographed using silica gel (hexane-EtOAc, 1:0 to 0:1) to provide 19 subfractions (A04080401–A04080419). Compound **4** (7.5 mg, t*_R_* 52 min) was obtained from A04080416 (24 mg) by preparative HPLC (YMC-Park PRO C18, MeOH-water, 7:3, 4 mL/min). Subfraction A04080408 (694 mg) was separated by RP-18 (MeOH-water, 6:4 to 1:0) to provide 10 subfractions (A0408040801–A0408040810). Subfraction A0408040803 (37 mg) was subjected to preparative HPLC (YMC-Park PRO C18, MeOH-water, 65:35, 5 mL/min) to yield **2** (1 mg, t*_R_* 92 min). Subfraction A0415 (1.66 g) was subjected to MPLC (hexane-EtOAc 95:5 to 0:1) to yield **10** (100 mg). Subfraction A041508 (68 mg) was purified by preparative HPLC (YMC-Actus Triart C18, MeOH-H2O, 6:4, 4 mL/min) to obtain **3** (0.9 mg, t*_R_* 149 min).

The aqueous extract (2.5 L) was subjected to column chromatography on Diaion HP-20 (MeOH-water 0:1 to 1:0) to provide five fractions (B01-B05). Fraction B04 (76 g) was subjected to a silica-gel column chromatography (CH_2_Cl_2_-MeOH 1:0 to 0:1) to afford **5** (2 g). Subfraction B0405 (2.1 g) was purified by MPLC (CH_2_Cl_2_-acetone 4:1 to 1:1, 5 mL/min), to afford 26 subfractions (B040501–B040526). Subfraction B040512 (320 mg) was subject to MPLC (CH_2_Cl_2_-acetone 3:1 to 2:1, 5 mL/min) to obtain 10 subfractions (B04051201–B04051210). Subfraction B04051205 (65 mg) was separated by Sephadex LH-20 column chromatography eluted with MeOH 100%, followed by purification via HPLC (YMC Pack Pro C18, MeOH-water 3:7, 4 mL/min) to provide **8** (4 mg, *t_R_* 57 min). Subfraction B0410 (10.3 g) was chromatographed over a silica gel column chromatography (CH_2_Cl_2_-MeOH 1:0 to 0:1) to yield 6 subfractions (B041001–B041006). Subfraction B041005 (2.8 g) was further separated by MPLC (CH_2_Cl_2_-acetone 1:0 to 0:1, 5 mL/min) to obtain eight subfractions (B04100501–B04100508). Subfraction B04100505 (675 mg) was separated by MPLC RP-18 (MeOH-water 9:1 to 1:0, 5 mL/min) and subsequently purified by HPLC (YMC-Pack Pro C18, MeOH-water 25:75, 2 mL/min), resulting in isolation of **6** (1.3 mg, t*_R_* 100 min).

*Methyl (10E,9R,16R)-16-acetoxy-9-hydroxyoctadeca-10,17-dien-12,14-diynoate* (**1**).

Brown amorphous solid; [α]_D_^20^ +3.3 (c 0.001, CHCl_3_); UV (MeOH) *λ*_max_ 215, 254, 268, 284 nm; ^1^H (CDCl_3_, 400 MHz) and ^13^C (CDCl_3_, 100 MHz), NMR data, see Table 2; HRESIMS *m*/*z* 383.1835 [M + Na]^+^ (calculated for C_21_H_28_NaO_5_, 383.1829).

*Methyl (10E,9R,16S)-9,16-dihydroxyoctadeca-10-en-12,14-diynoate* (**2**).

Brown amorphous solid; [α]_D_^20^ −18.0 (c 0.001, MeOH); UV (MeOH) *λ*_max_ 215, 254, 268, 283 nm; ^1^H (DMSO-*d*_6_, 400 MHz) and ^13^C (DMSO-*d*_6_, 100 MHz), NMR data, see Table 2; HRESIMS m/z 338.3419 [M + H_2_O]^+^ (calculated for C_19_H_30_O_5_, 338.2088).

*Methyl (10Z,9R,16S)-9,16-dihydroxyoctadeca-10,17-dien-12,14-diynoate* (**3**).

Brown amorphous solid; [α]_D_^20^ −18.2 (c 0.001, MeOH); UV (MeOH) *λ*_max_ 215, 254, 268, 283 nm; ^1^H (DMSO-*d*_6_, 400 MHz) and ^13^C (DMSO-*d*_6_, 100 MHz), NMR data, see Table 2; HRESIMS m/z 336.2172 [M + H_2_O]^+^ (calculated for C_19_H_28_O_5_, 336.1931).

### 3.4. Conformational Search and DP4+ Calculation for ***1***–***4***

Conformational searches for **1**–**4** were performed using MacroModel (version 11.9, Schrödinger LLC, New York, NY, USA) interfaced with Maestro (version 11.5, Schrödinger LLC) in a Merck molecular force field (MMFF94) in the gas phase. The conformers of **1**, **2**, and **4** were further geometrically optimized via density functional theory calculations made using TmoleX 4.3.2 and Turbomole 7.2 (COSMOLogic GmbH, Leverkusen, Germany) at the B3-LYP/def-SV(P) level in the gas phase, and the conformers of **3** were calculated at the CAM-B3LYP/def-SV(P) level. Optimized stable conformers with a Boltzmann population > 1% were selected for NMR chemical shift calculation with the basis set at 6–311G(d.p.) and the B3LYP functional level in the gas phase. The calculated isotropic magnetic shielding constants (σ) were Boltzmann distribution averaged according to their free energies. The results were then put into Sarotti’s Excel spreadsheet (version 1.3.2) for calculation of the DP4+ probability to determine the best fit [80].

### 3.5. ECD Calculation

All conformers were geometrically optimized at the B3-LYP/def-SV(P) level in the gas phase. Stable conformers with a Boltzmann population of > 1% were selected for the ECD calculation. ECD spectra were simulated using the TD-DFT method and applying the B3LYP/def-SVP level in the gas phase. The overall ECD curves were all weighted by the Boltzmann distribution and produced by SpecDis 1.71 software with a sigma/gamma value of 0.24 eV and UV corrections of −18 nm (**1**), −10 nm (**2**), −15 nm (**3**), and −15 nm (**4**) to facilitate comparison with the experimental data [81].

### 3.6. Molecular Docking

The crystal structures of AMPK and CTSS were obtained from the RCSB PDB website [PDB ID: 2Y94 (AMPK) and 6YYR (CTSS)] (https://www.rcsb.org/, accessed on 1 March 2024). The 3D structures of AICAR and LY3000328 were acquired from the PubChem website (https://pubchem.ncbi.nlm.nih.gov/, accessed on 1 March 2024). Four programs were used for the docking simulations: AutoDock Vina 1.1.2 (https://vina.scripps.edu/, accessed on 1 March 2024), AutoDock 4.2.6 (https://autodock.scripps.edu/, accessed on 1 March 2024), LeDock (http://www.lephar.com/software.htm, accessed on 1 March 2024), and Dock 6.10 (https://dock.compbio.ucsf.edu/DOCK_6/index.htm, accessed on 1 March 2024). Docking preparation was conducted to add hydrogens and assign charges to the compounds in UCSF Chimera (https://www.rbvi.ucsf.edu/chimera/, accessed on 1 March 2024). A pharmacophore analysis was conducted using LigandScout 4.0 (Inte:Ligand, Vienna, Austria, (http://www.inteligand.com/ligandscout/, accessed on 1 March 2024) to explore possible receptor and ligand interactions.

### 3.7. Cell Treatment Experiments

HepG2 cells (human liver cancer cell line) were purchased from the American Type Culture Collection (Manassas, VA, USA). The cells were cultured in Dulbecco’s modified Eagle medium (Welgene, Gyeongsan, Gyeongsangbuk-do, Republic of Korea) supplemented with 10% heat-inactivated (56 °C for 30 min) fetal bovine serum (Welgene), 100 U/mL penicillin, and 100 μg/mL streptomycin (Welgene). The cells were maintained at 37 °C in a humidified atmosphere with 5% CO_2_. The medium was replaced with fresh medium after 2 days to remove non-adherent cells or cell debris. To evaluate the effects of **7** on lipid accumulation, HepG2 cells were pretreated with 2.6 μM of **7** for 3 h before treatment with BSA-conjugated palmitate (500 μM) for 24 h.

### 3.8. Protein Extraction and Western Blotting

To lyse the cells and extract proteins, the cells were rinsed with phosphate-buffered saline (PBS, Gibco, Grand Island, NY, USA) and collected using cold PBS. Total protein was extracted from the cells using cold RIPA buffer (Biosesang, Seongnam, Republic of Korea) containing a protease inhibitor cocktail (GenDEPOT, Katy, TX, USA) according to the manufacturer’s protocol. Whole-cell lysates (10–40 μg of protein) were prepared by heating at 98 °C for 5 min in a gel-loading buffer (0.3125 M Tris-HCl pH 6.8, 2% SDS, 5% 2-mercaptoethanol, 0.05% bromophenol blue, and 25% glycerol) at a 4:1 volume ratio. Protein samples were quantified using Pierce™ BCA protein assay kits (Thermo Scientific, Waltham, MA, USA). The proteins were then separated using SDS-PAGE and transferred onto PVDF membranes (Millipore, Burlington, MA, USA) with a Bio-Rad western system. The membranes were blocked for 45 min with 5% non-fat milk in TBS (50 mM Tris, 150 mM NaCl, pH 7.6) containing 0.1% Tween 20 (TBS-T). After blocking, the membranes were washed five times with TBS-T for 15 min each and incubated overnight at 4 °C with primary antibodies at dilutions of 1:1,000. The next day, the membranes were washed four times with TBS-T for 15 min each and then incubated for 1 h at 25 °C with horseradish peroxidase (HRP)-conjugated secondary antibodies (1:10,000). The immunoblots were visualized using chemiluminescent HRP substrate (Advansta, San Jose, CA, USA) and a Davinchchemi CAS-400 (Davinch-K, Seoul, Republic of Korea), according to the manufacturer’s instructions.

### 3.9. Transfection and Luciferase Assay

To examine the activity of **7** in cells, PPAR-α luciferase assays were performed. Twenty-four hours after transfection with Lipofectamine 3000 transfection reagent (Invitrogen, Grand Island, NY, USA) and a PPAR-α promoter-LUC plasmid (0.1 μg), cells were treated with **7** (2.6 μM) and WY14643 (20 μM) for 24 h. Luciferase activity was then detected using a One-Glo luciferase assay system (Promega, Madison, WI, USA) and measured using a luminometer (Berthold Technologies GmbH & Co., Bad Wildbad, Germany).

### 3.10. CTSS Activity Assay

The CTSS activity assay was performed as follows: Assay buffer was prepared from 50 mM potassium phosphate buffer (pH 6.5), 50 mM NaCl, 2 mM EDTA, 0.01% Triton X-100, and 0.5 mM DTT. To that buffer, 2 µL of sample was added to meet 100 µL of total solution. Subsequently, 20 µM Z-VVR-AMC (Chinese Peptide Company, Hangzhou, China) was added as the CTSS substrate. The reaction was monitored kinetically for 30 min, with fluorescence measurements taken at an excitation wavelength of 380 nm and an emission wavelength of 460 nm.

### 3.11. Reagents

All antibodies were purchased from Santa Cruz Biotechnology (Dallas, TX, USA), Abcam (Cambridge, MA, USA), or Cell Signaling Technology, Inc. (Beverly, MA, USA). The primary antibodies were as follows: anti-PPAR-α (sc-398394), anti-Sirt1 (ab13749), anti-α-tubulin (sc-52**8**6), and anti-AMPK (Thr172) (#2535). Palmitic acid (P9767) was obtained from Sigma-Aldrich (Milwaukee, WI, USA). WY14643, a PPAR-α agonist, was purchased from MedChemExpress (Monmouth Junction, NJ, USA).

## 4. Conclusions

In this work, 10 compounds were isolated from *D. morbifera*: three new polyacetylenes (**1**–**3**) and seven known compounds (**4**–**10**). Their structures were determined using 1D and 2D NMR spectroscopy and HRESIMS, and the absolute configurations of **1**–**4** were established through ECD calculations and DP4+ simulations. Compound **4** is a known substance, but its absolute configuration had not been determined before. Based on literature search to find therapeutic targets, all the isolated compounds were evaluated using in silico molecular docking to determine their binding affinity with AMPK and CTSS receptors. Among them, **7** exhibited the highest binding energy for both AMPK and CTSS. In the in vitro assays, **7** stimulated PPAR-α luciferase activity and increased the protein levels of AMPK, PPAR-α, and Sirt1, indicating its role as a PPAR-α activator. However, in the in vitro CTSS assays, **7** exhibited no significant inhibition of CTSS. Based on the biological activity of **7** as a PPAR-α activator, it can be a potential candidate to relieve hepatic lipogenesis, adipose differentiation, and glucose metabolism in muscle by activating the AMPK pathway. Nevertheless, further research is needed to elucidate the specific mechanisms through which PPAR-α activation contributes to those diseases. This study expands our chemical and biological understanding of *D. morbifera* and will provide information to future investigators.

## Figures and Tables

**Table 1:** ^1^H NMR (400 MHz) and ^13^C NMR (100 MHz) of **1** in CDCl_3_, and **2** and **3** in DMSO-*d*_6_.

Position	1		2		3	
	*δ*_H_	*δ*_C_	*δ*_H_	*δ*_C_	*δ*_H_	*δ*_C_
1		174.3		173.3		173.3
2	2.30 (2H, t, *J* = 7.5 Hz)	34.1	2.28 (2H, t, *J* = 7.4 Hz)	33.2	2.28 (2H, t, *J* = 7.4 Hz)	33.2
3	1.62 (2H, m)	24.9	1.50 (2H, m)	24.3	1.50 (2H, m)	24.3
4	1.31 (2H, m)	29.0 ^a^	1.24 (2H, m)	28.3	1.25 (2H, m)	28.3
5	1.31 (2H, m)	29.1^a^	1.24 (2H, m)	28.7 ^a^	1.25 (2H, m)	28.5 ^a^
6	1.25 (2H, m)	29.2	1.24 (2H, m)	28.5 ^a^	1.25 (2H, m)	28.6 ^a^
7	1.32 (2H, m)	25.1	1.26 (2H, m)	24.7	1.26 (2H, m)	24.5
8	1.53 (2H, m)	36.8	1.37 (2H, m)	36.3	1.49 (1H, m) 1.34 (1H, m)	36.4
9	4.20 (1H, m)	72.0	4.03 (1H, m)	69.8	4.35 (1H, m)	68.5
10	6.34 (1H, dd, *J* = 15.9, 5.7 Hz)	150.2	6.39 (1H, dd, *J* = 15.8, 5.1 Hz)	152.5	6.10 (1H, dd, *J* = 11.1, 8.7 Hz)	152.5
11	5.76 (1H, ddd, *J* = 15.9, 1.6, 0.8 Hz)	107.9	5.77 (1H, d, *J* = 15.8 Hz)	105.9	5.66 (1H, d, *J* = 11.1 Hz)	106.2
12		77.7		76.9		77.1
13		73.5		73.1		75.2
14		71.5		67.6		68.4
15		77.2		85.3		84.2
16	5.96 (1H, dq, *J* = 5.8, 1.0 Hz)	64.7	4.28 (1H, t, *J* = 6.5 Hz)	62.0	4.93 (1H, d, *J* = 5.4 Hz)	61.7
17	5.88 (1H, ddd, *J* = 16.9, 10.3, 5.7 Hz)	132.1	1.58 (2H, m)	30.3	5.88 (1H, ddd, *J* = 17.0, 10.1, 5.4 Hz)	137.3
18	*trans*: 5.55 (1H, dt, *J* = 16.9, 1.1 Hz) *cis*: 5.35 (1H, dt, *J* = 10.3, 1.1 Hz)	119.6	0.90 (3H, t, *J* = 7.4 Hz)	9.4	*trans*: 5.33 (1H, dt, *J* = 17.1, 1.5 Hz) *cis*: 5.16 (1H, dt, *J* = 10.1, 1.5 Hz)	115.5
1-OCH_3_	3.67 (3H, s)	51.5	3.58 (3H, s)	51.1	3.58 (3H, s)	51.0
16-OCOCH_3_		169.5				
16-OCOCH_3_	2.11 (3H, s)	20.9				

^a^ Assignments in the same column are interchangeable.

**Table 2:** Docking energy (Kcal/mol) of **1**–**10** and AICAR ^a^ with AMPK.

Compound	AutoDock Vina	AutoDock 4	LeDock	Dock 6
AICAR (Control)	−6.3	−7.4	−6.0	−33.5
**1**	−5.8	−5.3	−3.5	−42.9
**2**	−5.7	−5.3	−4.2	−38.3
**3**	−5.8	−5.8	−4.4	−39.5
**4**	−5.8	−5.5	−4.1	−35.6
**5**	−6.8	−7.2	−5.2	−37.8
**6**	−6.8	−8.7	−6.0	−55.5
**7**	−8.9	−11.4	−7.0	−40.6
**8**	−6.6	−6.6	−4.4	−46.3
**9**	−6.5	−9.3	−3.3	−28.5
**10**	−7.6	−7.9	−4.5	−43.8

^a^ AICAR, 5-amino-4-imidazolecarboxamide ribonucleoside.

**Table 3:** Docking energy (Kcal/mol) of **1**–**10** and LY3000328 with CTSS.

Compound	AutoDock Vina	AutoDock 4	LeDock	Dock 6
LY3000328 (Control)	−7.2	−8.1	−5.6	−127.0
**1**	−5.2	−4.6	−4.0	−39.5
**2**	−5.7	−4.6	−4.2	−42.6
**3**	−5.7	−5.7	−4.5	−44.0
**4**	−5.6	−4.6	−4.2	−43.6
**5**	−6.3	−6.9	−5.8	−39.3
**6**	−6.5	−9.1	−5.8	−50.5
**7**	−8.1	−10.3	−6.1	−42.2
**8**	−6.5	−5.9	−4.5	−36.5
**9**	−8.5	−9.4	−3.8	−33.1
**10**	−6.9	−7.3	−4.6	−42.7

## Data Availability

Data will be made available on request.

## Supplementary Materials

The following supporting information can be downloaded at: https://www.mdpi.com/article/10.3390/molecules29245942/s1. Figure S1. UV spectrum of 1; Figure S2. (+)-HR-ESI-MS spectrum of 1; Figure S3. 1H NMR spectrum of 1 (400 MHz, CDCl3); Figure S4. 13C NMR spectrum of 1 (100 MHz, CDCl3); Figure S5. DEPT135 spectrum of 1 (100 MHz, CDCl3); Figure S6. HSQC spectrum of 1 (1H: 400 MHz, 13C: 100 MHz, CDCl3); Figure S7. HMBC spectrum of 1 (1H: 400 MHz, 13C: 100 MHz, CDCl3); Figure S187. 1H-1H COSY spectrum of 1 (400 MHz, CDCl3); Figure S9. NOESY spectrum of 1 (400 MHz, CDCl3); Figure S10. UV spectrum of 2; Figure S11. (+)-HR-ESI-MS spectrum of 2; Figure S12. 1H NMR spectrum of 2 (400 MHz, DMSO-d6); Figure S13. 13C NMR spectrum of 2 (100 MHz, DMSO-d6); Figure S14. DEPT135 spectrum of 2 (100 MHz, DMSO-d6); Figure S15. HSQC spectrum of 2 (1H: 400 MHz, 13C: 100 MHz, DMSO-d6); Figure S16. HMBC spectrum of 2 (1H: 400 MHz, 13C: 100 MHz, DMSO-d6); Figure S17. 1H-1H COSY spectrumof 2 (400 MHz, DMSO-d6); Figure S18. NOESY spectrum of 2 (400 MHz, DMSO-d6); Figure S19. UV spectrum of 3; Figure S20. (+)-HR-ESI-MS spectrum of 3; Figure S21. 1H NMR spectrum of 3 (400 MHz, DMSO-d6); Figure S22. 13C NMR spectrum of 3 (100 MHz, DMSO-d6); Figure S23. DEPT135 spectrum of 3 (100 MHz, DMSO-d6); Figure S24. HSQC spectrum of 3 (1H: 400 MHz, 13C: 100 MHz, DMSO-d6); Figure S25. HMBC spectrum of 3 (1H: 400 MHz, 13C: 100 MHz, DMSO-d6); Figure S26. 1H-1H COSY spectrum of 3 (400 MHz, DMSO-d6); Figure S27. NOESY spectrum of 3 (400 MHz, DMSO-d6); Figure S28. Linear correlation plots between calculated and experimental 1H and 13C NMR chemical shift values for two potential diastereomers (9R*16S* and 9R*16R*) of 1; Figure S29. DP4+ probability for diastereomer 1 (9R*16S*) and diastereomer 2 (9R*16R*) of 1; Figure S30. Linear correlation plots between calculated and experimental 1H and 13C NMR chemical shift values for two potential diastereomers (9R*16S* and 9R*16R*) of 2; Figure S31. DP4+ probability for diastereomer 1 (9R*16S*) and diastereomer 2 (9R*16R*) of 2; Figure S32. Linear correlation plots between calculated and experimental 1H and 13C NMR chemical shift values for two potential diastereomers (9R*16S* and 9R*16R*) of 3; Figure S33. DP4+ probability for diastereomer 1 (9R*16S*) and diastereomer 2 (9R*16R*) of 3; Figure S34. Linear correlation plots between calculated and experimental 1H and 13C NMR chemical shift values for two potential diastereomers (9R*16S* and 9R*16R*) of 4; Figure S35. DP4+ probability for diastereomer 1 (9R*16S*) and diastereomer 2 (9R*16R*) of 4; Table S1. Energy analysis for diastereomer 1 (9R*16S*) and diastereomer 2 (9R*16R*) of 1; Table S2. Calculated (calcd.) and experimental (exp.) 13C chemical shift values for diastereomer 1 (9R*16S*) and diastereomer 2 (9R*16R*) of 1; Table S3. Calculated (calcd.) and experimental (exp.) 1H chemical shift values for diastereomer 1 (9R*16S*) and diastereomer 2 (9R*16R*) of 1; Table S4. Energy analysis for diastereomer 1 (9R*16S*) and diastereomer 2 (9R*16R*) of 2; Table S5. Calculated (calc.) and experimental (exp.) 13C chemical shift values for diastereomer 1 (9R*16S*) and diastereomer 2 (9R*16R*) of 2; Table S6. Calculated (calc.) and experimental (exp.) 1H chemical shift values for diastereomer 1 (9R*16S*) and diastereomer 2 (9R*16R*) of 2; Table S7. Energy analysis for diastereomer 1 (9R*16S*) and diastereomer 2 (9R*16R*) of 3; Table S8. Calculated (calc.) and experimental (exp.) 13C chemical shift values for diastereomer 1 (9R*16S*) and diastereomer 2 (9R*16R*) of 3; Table S9. Calculated (calc.) and experimental (exp.) 1H chemical shift values for diastereomer 1 (9R*16S*) and diastereomer 2 (9R*16R*) of 3; Table S10. Energy analysis for diastereomer 1 (9R*16S*) and diastereomer 2 (9R*16R*) of 4; Table S11. Calculated (calc.) and experimental (exp.) 13C chemical shift values for diastereomer 1 (9R*16S*) and diastereomer 2 (9R*16R*) of 4; Table S12. Calculated (calc.) and experimental (exp.) 1H chemical shift values for diastereomer 1 (9R*16S*) and diastereomer 2 (9R*16R*) of 4.
