# Supplementary material for: Identification of New Polyacetylenes from Dendropanax morbifera with PPAR-α Activity Study
Source: Molecules. 2024 Dec 16;29(24):5942. doi: 10.3390/molecules29245942 (PMC11677830; doi:10.3390/molecules29245942)
Supplement: Supplementary file 1 [file molecules-29-05942-s001.zip › molecules-3302005-supplementary.pdf]

Supplementary Data

Identification of New Polyacetylenes from *Dendropanax morbifera* with PPAR-α Activity Study

Dong-Lan Piao [1,†], Isoo Youn [1,†], Thanh-Hau Huynh [2], Hyun-Woo Kim [3], Sang-Gyun Noh [3], Hae-Young Chung [3], Dong-Chan Oh [2] and Eun-Kyoung Seo [1,*]

1 Graduate School of Pharmaceutical Sciences, College of Pharmacy, Ewha Womans University, Seoul 03760, Republic of Korea; parkdl@ewhain.net (D.P.); iyoun@ewha.ac.kr (I.Y.)
2 Natural Products Research Institute, College of Pharmacy, Seoul National University, Seoul 08826, Republic of Korea; 2019-22632@snu.ac.kr (T.-H.H.); dongchanoh@snu.ac.kr (D.-C.O.)
3 Department of Pharmacy and Research Institute for Drug Development, College of Pharmacy, Pusan National University, Busan 46241, Republic of Korea; khw124124@naver.com (H.W.K.); rskrsk92@pusan.ac.kr (S.G.N.); hyjung@pusan.ac.kr (H.Y.C.)
* Correspondence: yuny@ewha.ac.kr; Tel.: +82-2-3277-3047
† These authors contributed equally to this work.

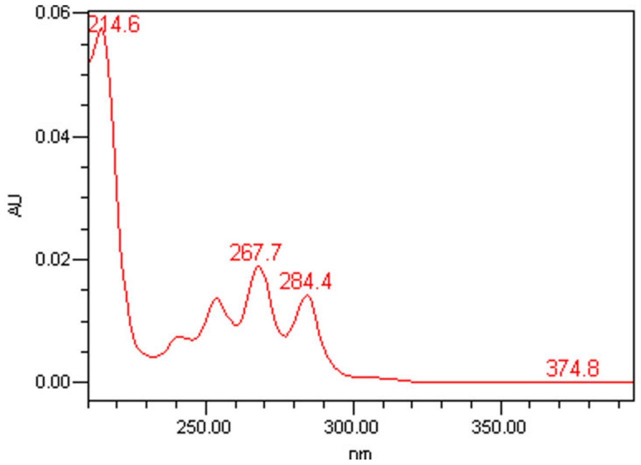

Figure S1. UV spectrum of **1**.

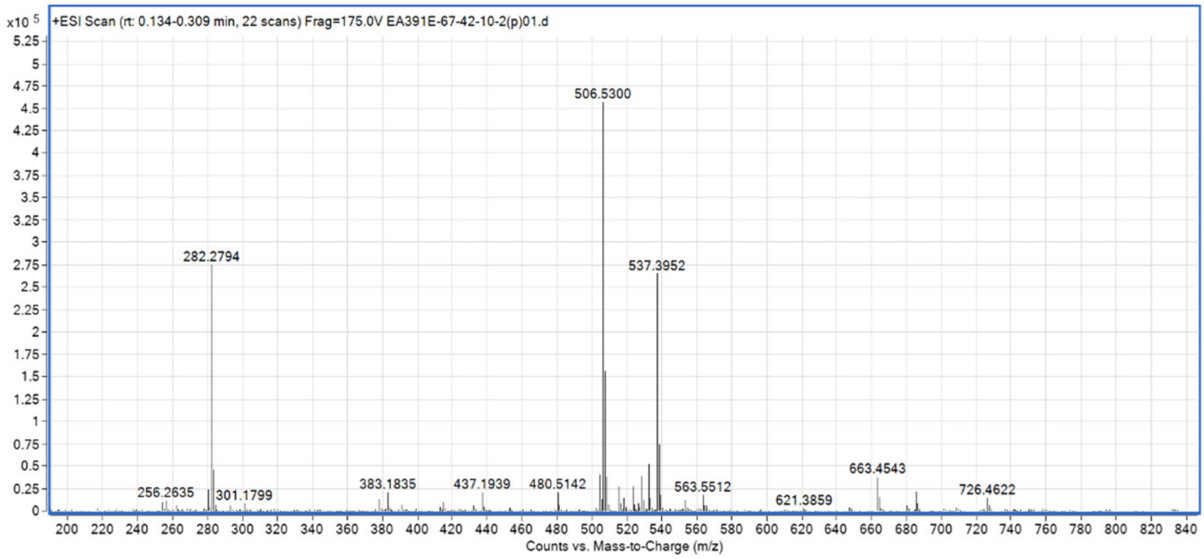

Figure S2. (+)-HR-ESI-MS spectrum of **1**.

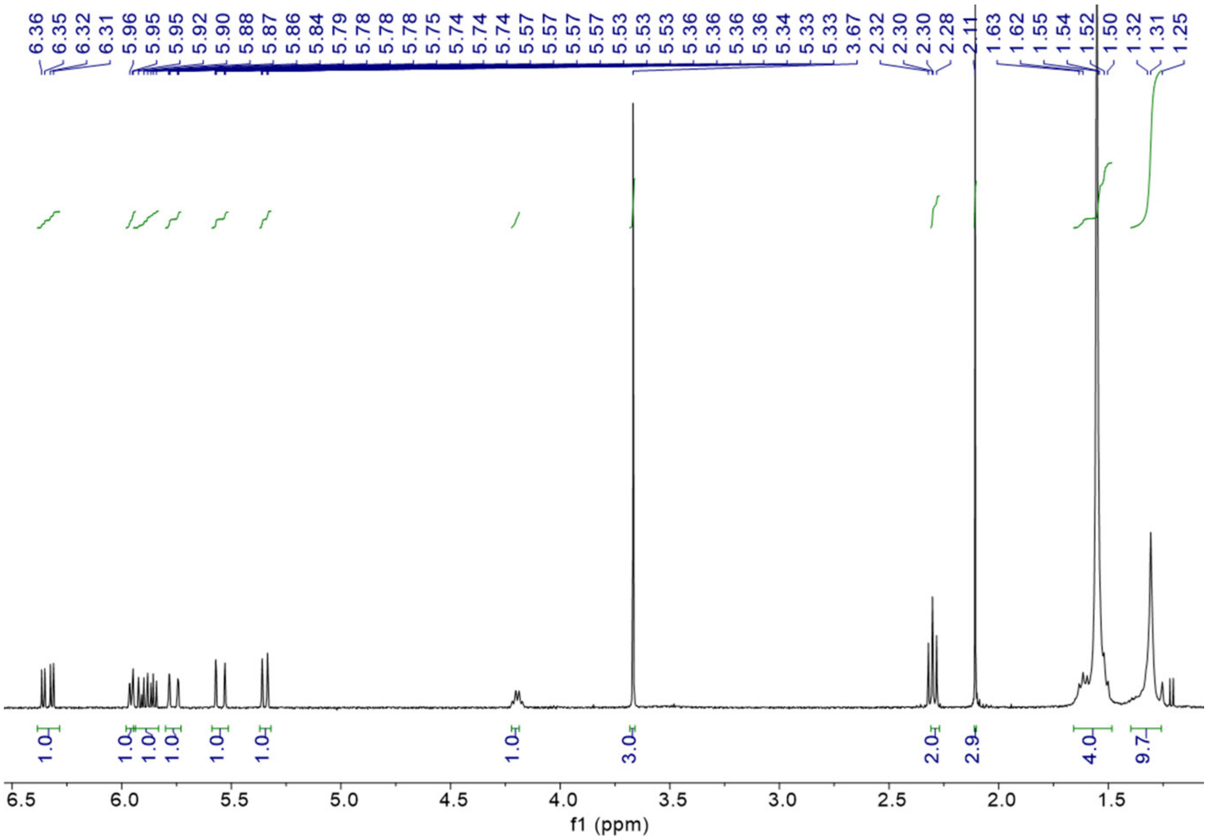

Figure S3. ^{1}H NMR spectrum of **1** (400 MHz, $CDCl_3$).

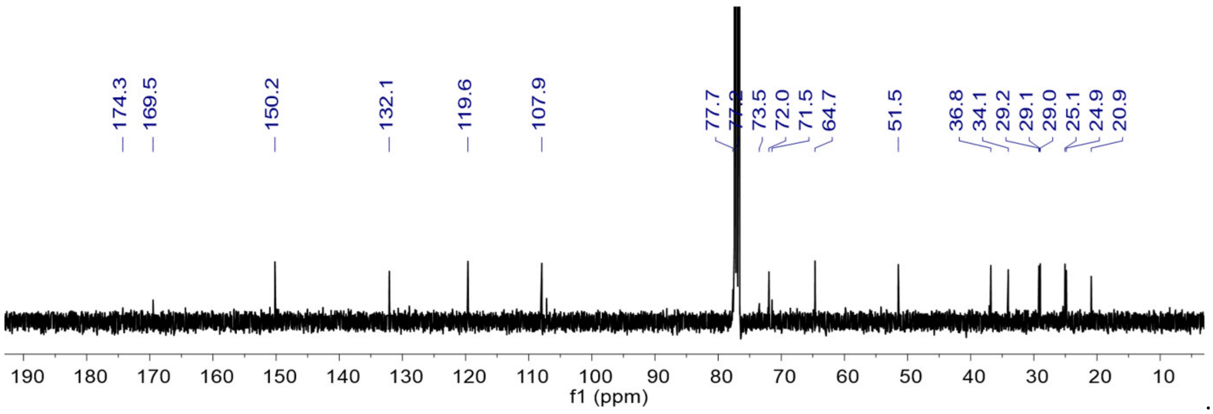

Figure S4. ^{13}C NMR spectrum of **1** (100 MHz, $CDCl_3$).

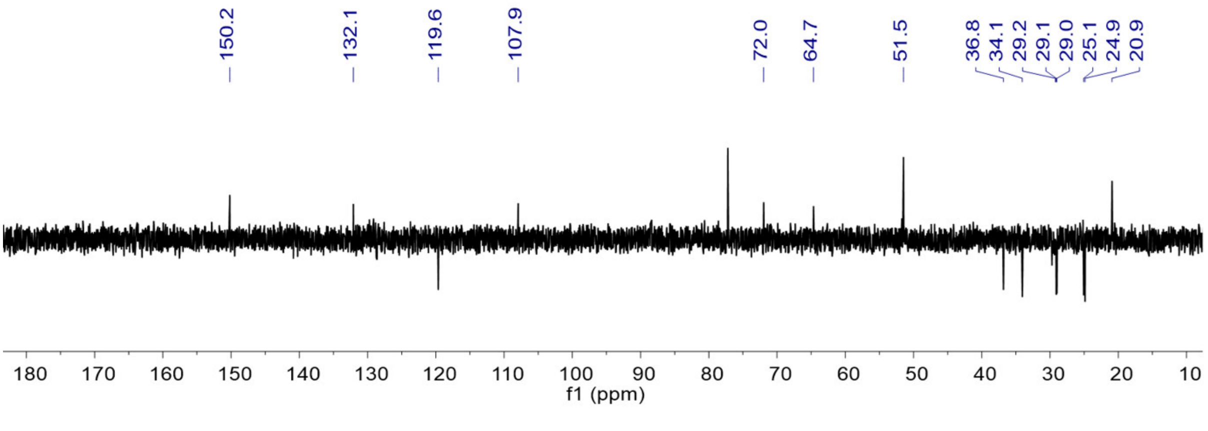

Figure S5. DEPT135 spectrum of **1** (100 MHz, $CDCl_3$).

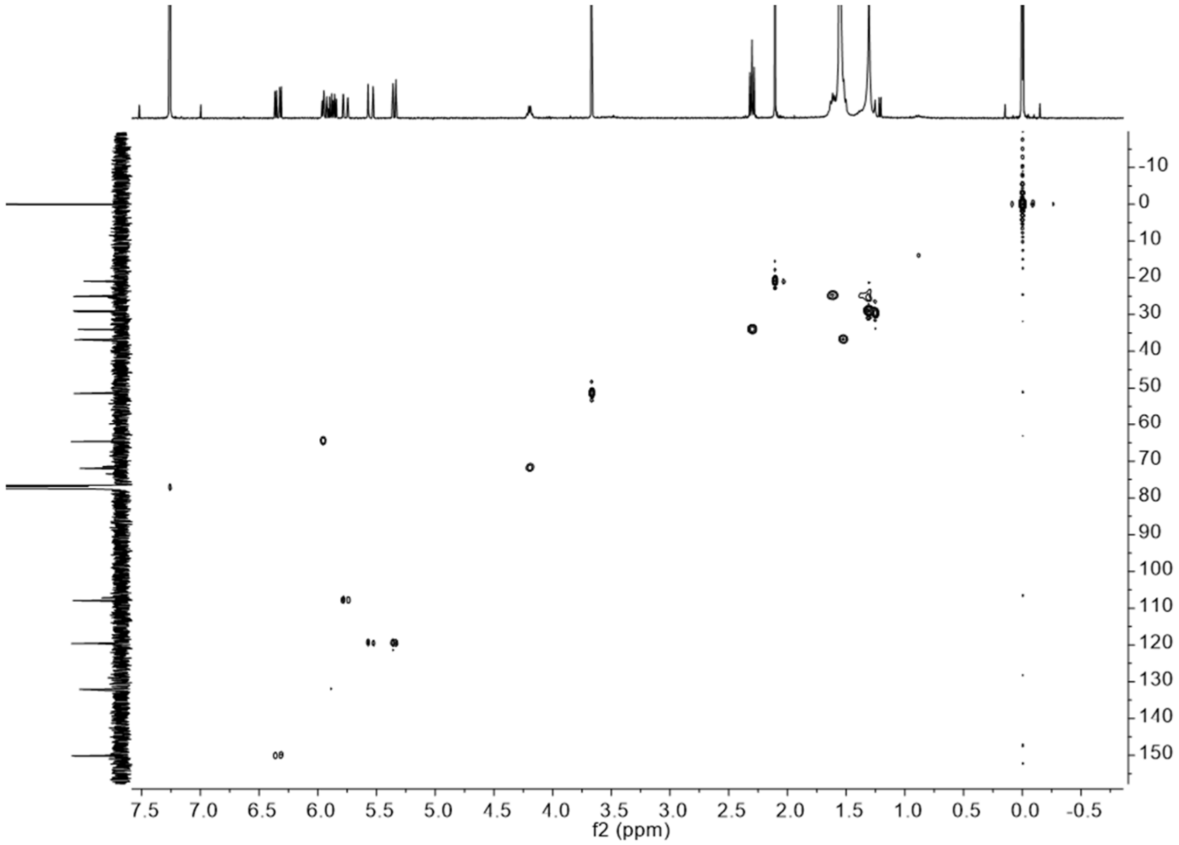

Figure S6. HSQC spectrum of **1** (^1H: 400 MHz, ^{13}C: 100 MHz, $CDCl_3$). .

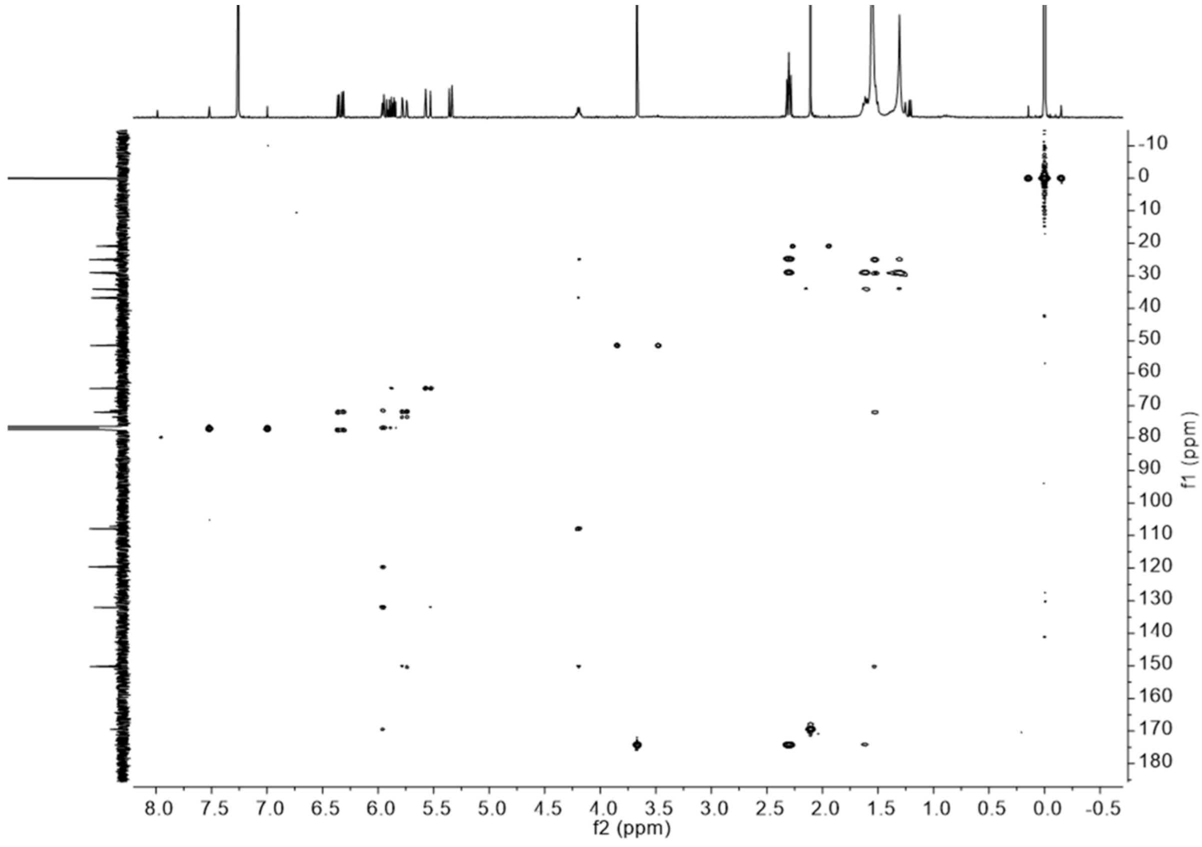

Figure S7. HMBC spectrum of **1** (^{1}H: 400 MHz, ^{13}C: 100 MHz, $CDCl_3$).

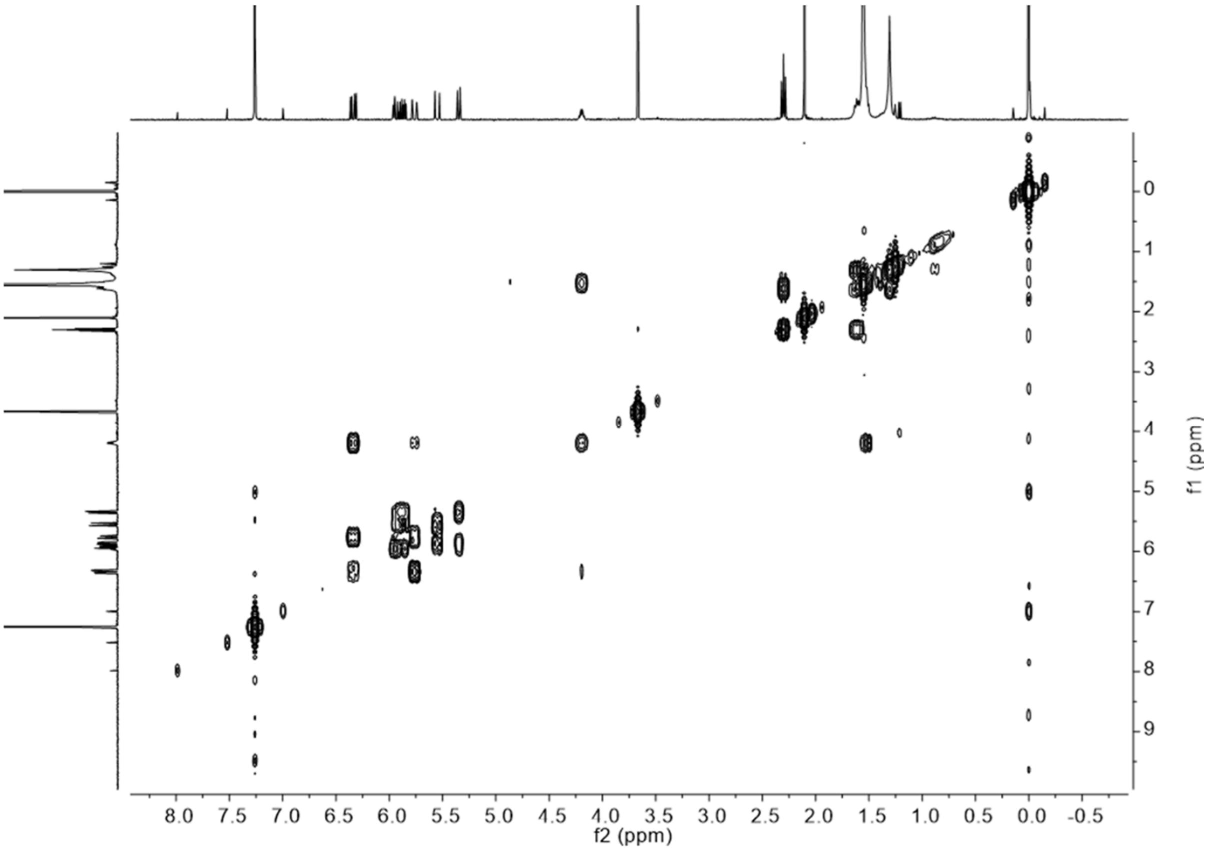

Figure S8. ^{1}H-^{1}H COSY spectrum of **1** (400 MHz, $CDCl_3$).

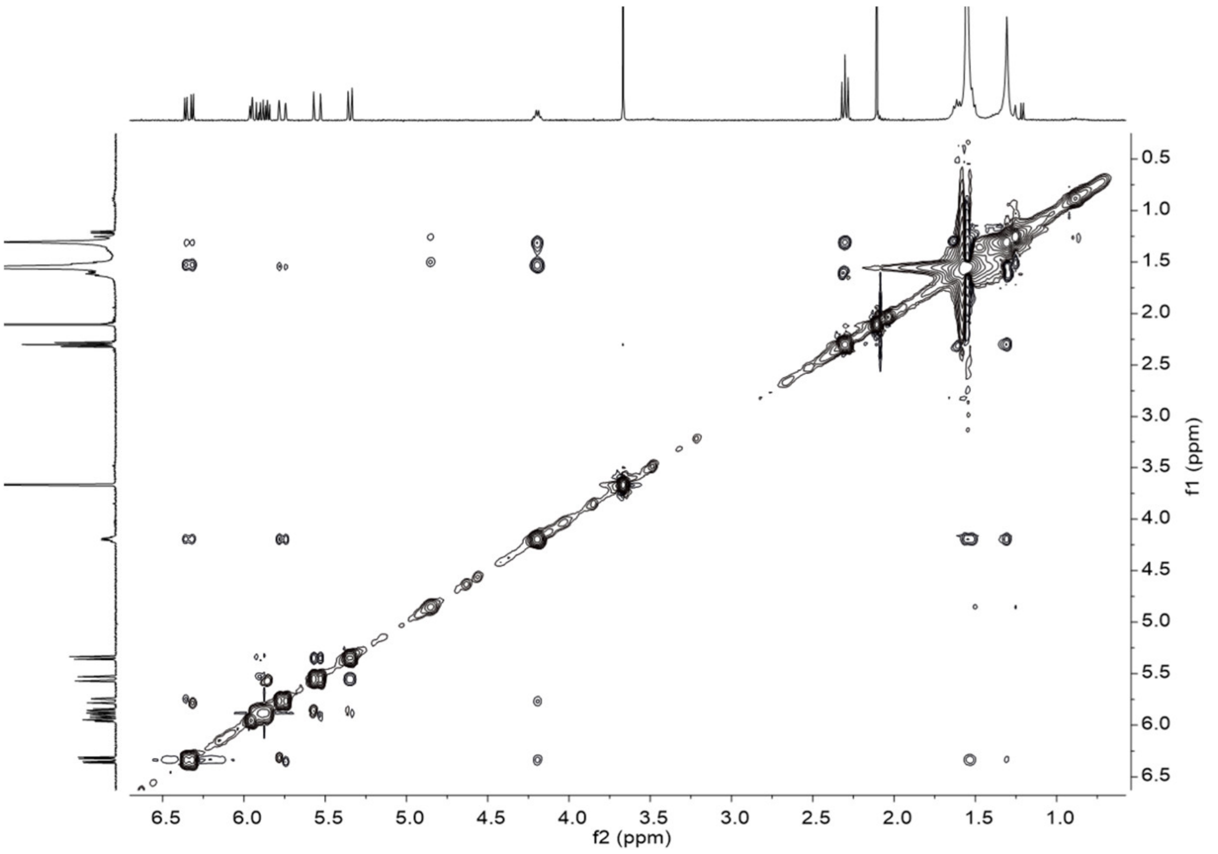

Figure S9. NOESY spectrum of **1** (400 MHz, $CDCl_3$).

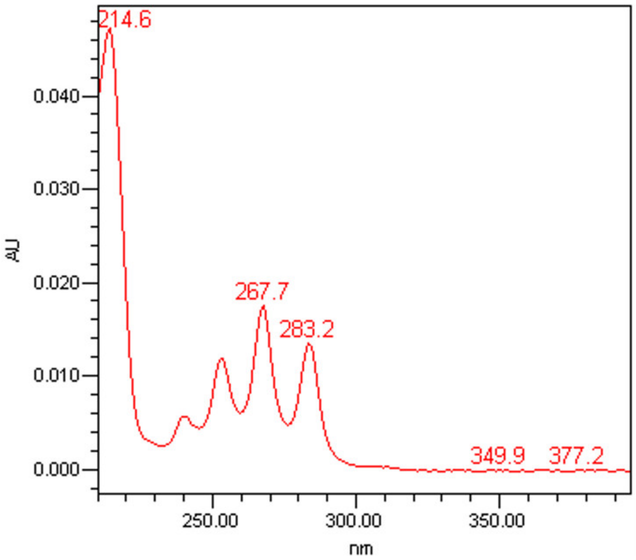

Figure S10. UV spectrum of **2**.

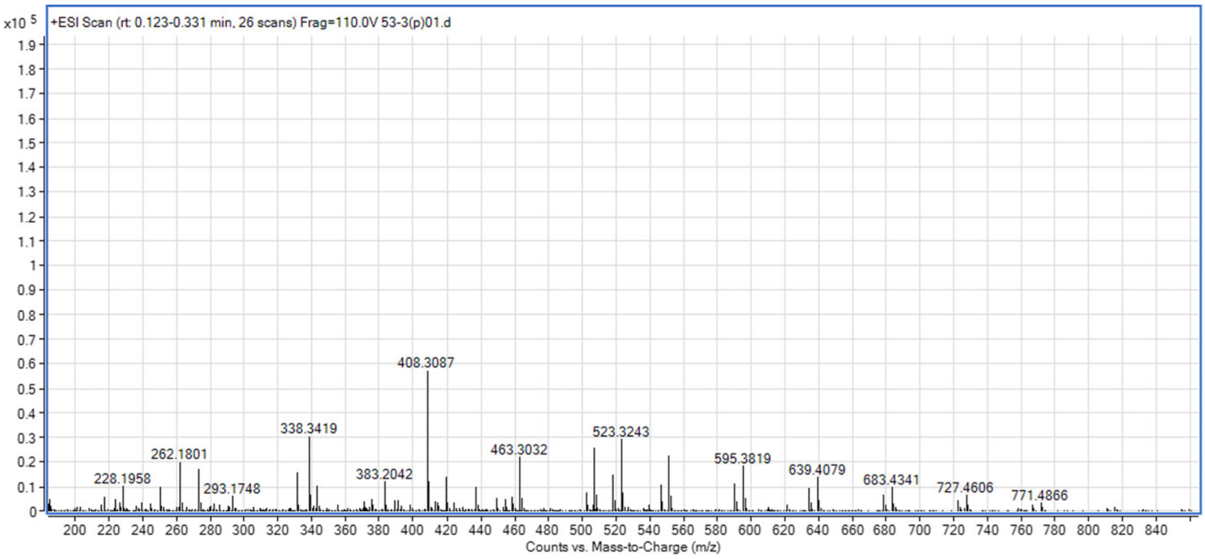

Figure S11. (+)-HR-ESI-MS spectrum of **2**.

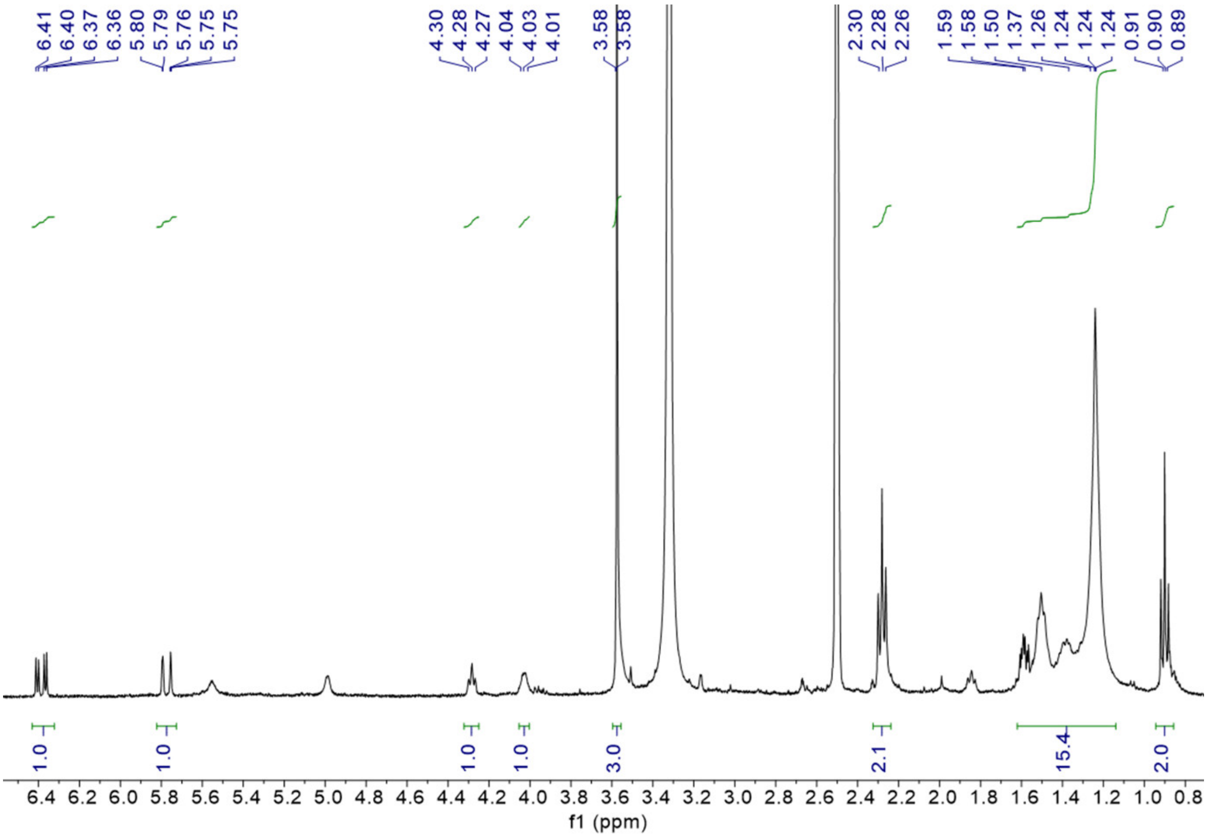

Figure S12. ^1H NMR spectrum of **2** (400 MHz, DMSO-d_6).

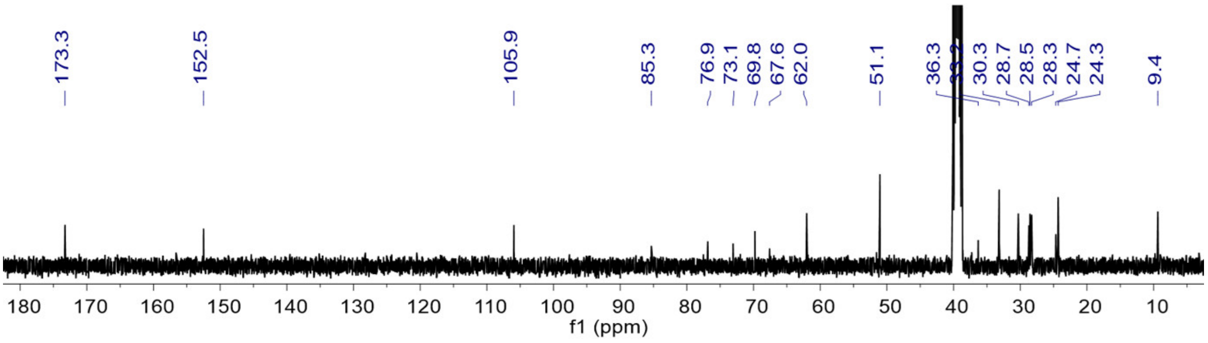

Figure S13. ^{13}C NMR spectrum of **2** (100 MHz, DMSO-d_6).

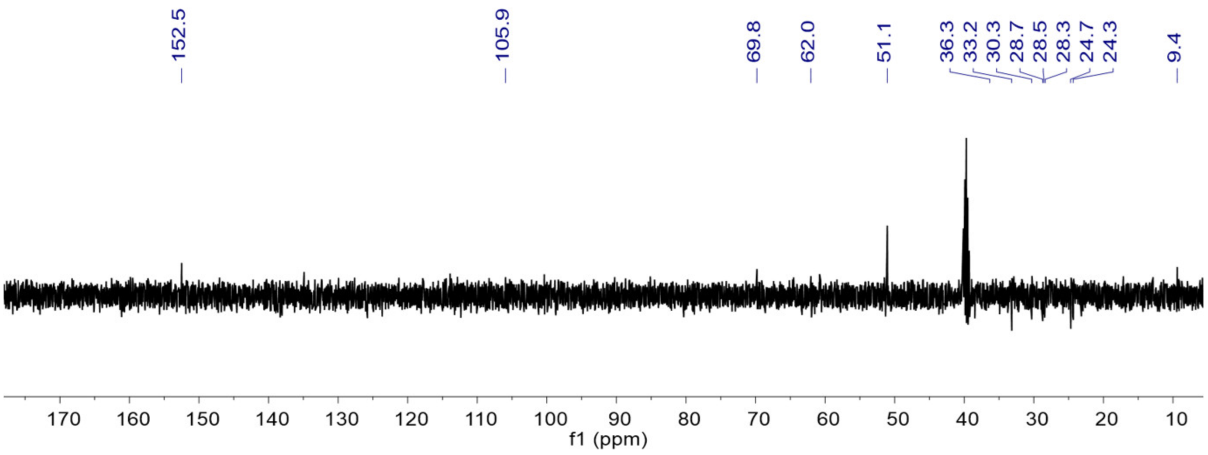

Figure S14. DEPT135 spectrum of **2** (100 MHz, DMSO-d_6).

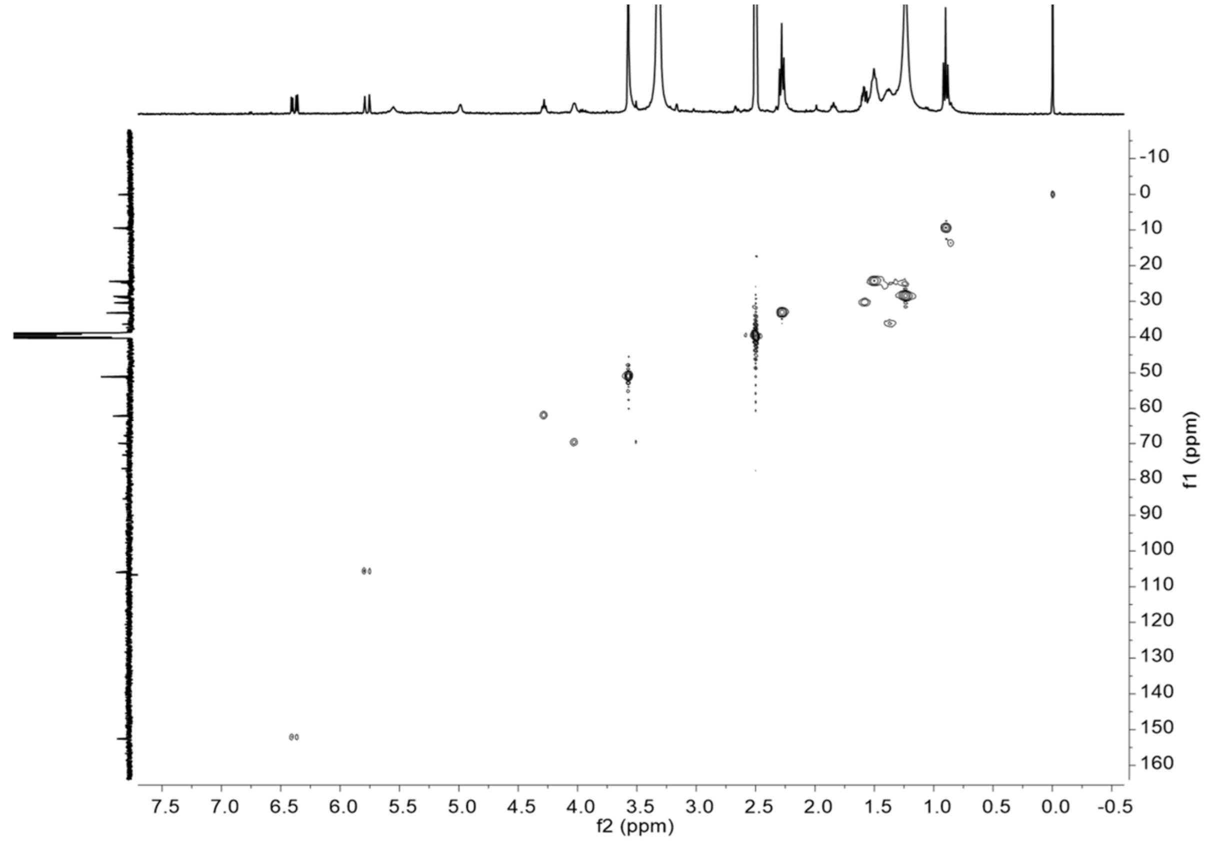

Figure S15. HSQC spectrum of **2** (^{1}H: 400 MHz, ^{13}C: 100 MHz, DMSO-d_6).

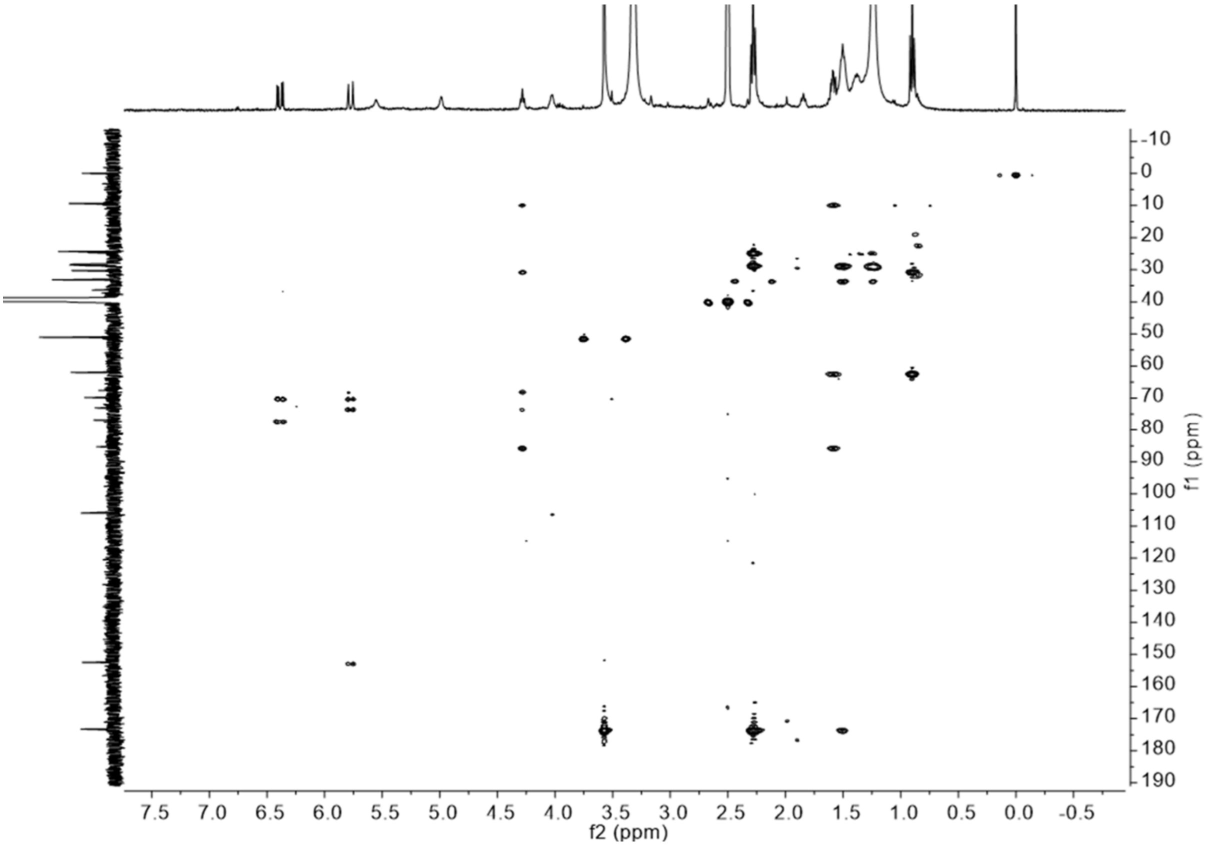

Figure S16. HMBC spectrum of **2** (^{1}H: 400 MHz, ^{13}C: 100 MHz, DMSO-d_6).

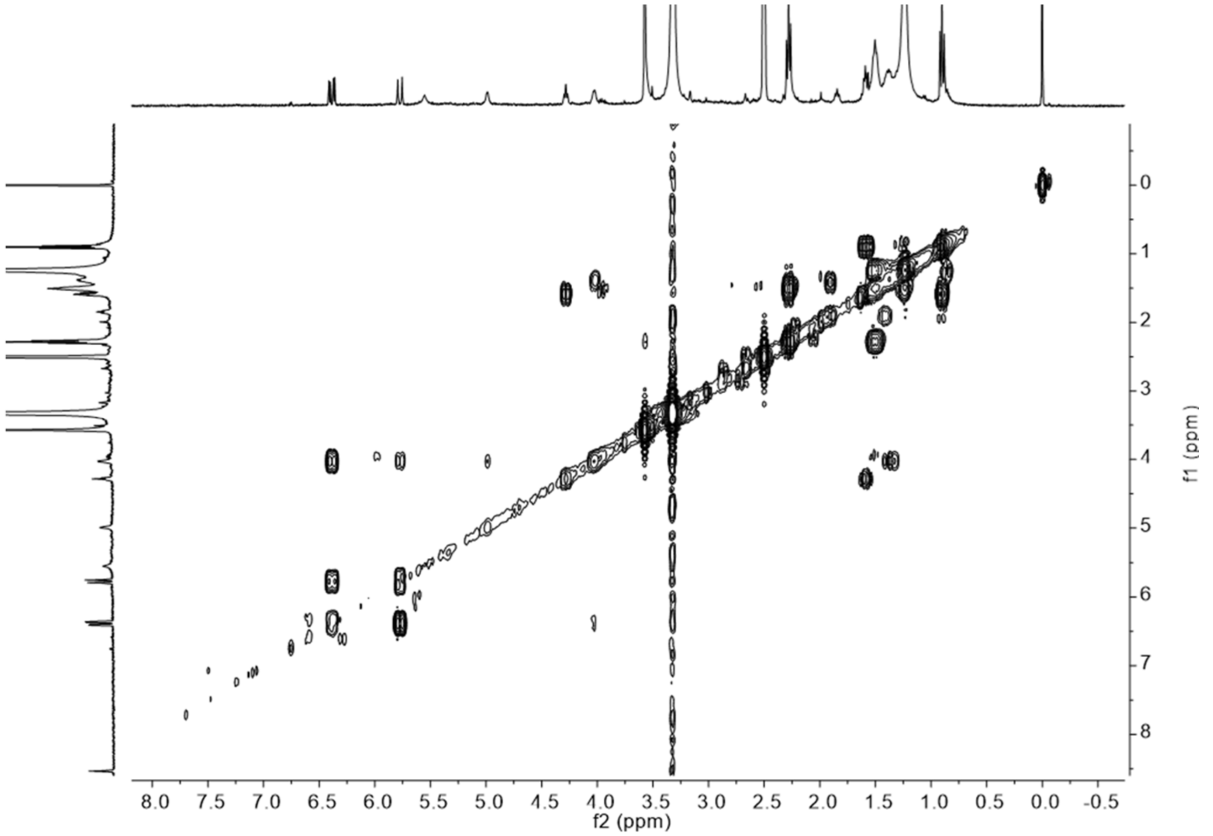

Figure S17. ^1H-^1H COSY spectrum of **2** (400 MHz, DMSO-d_6).

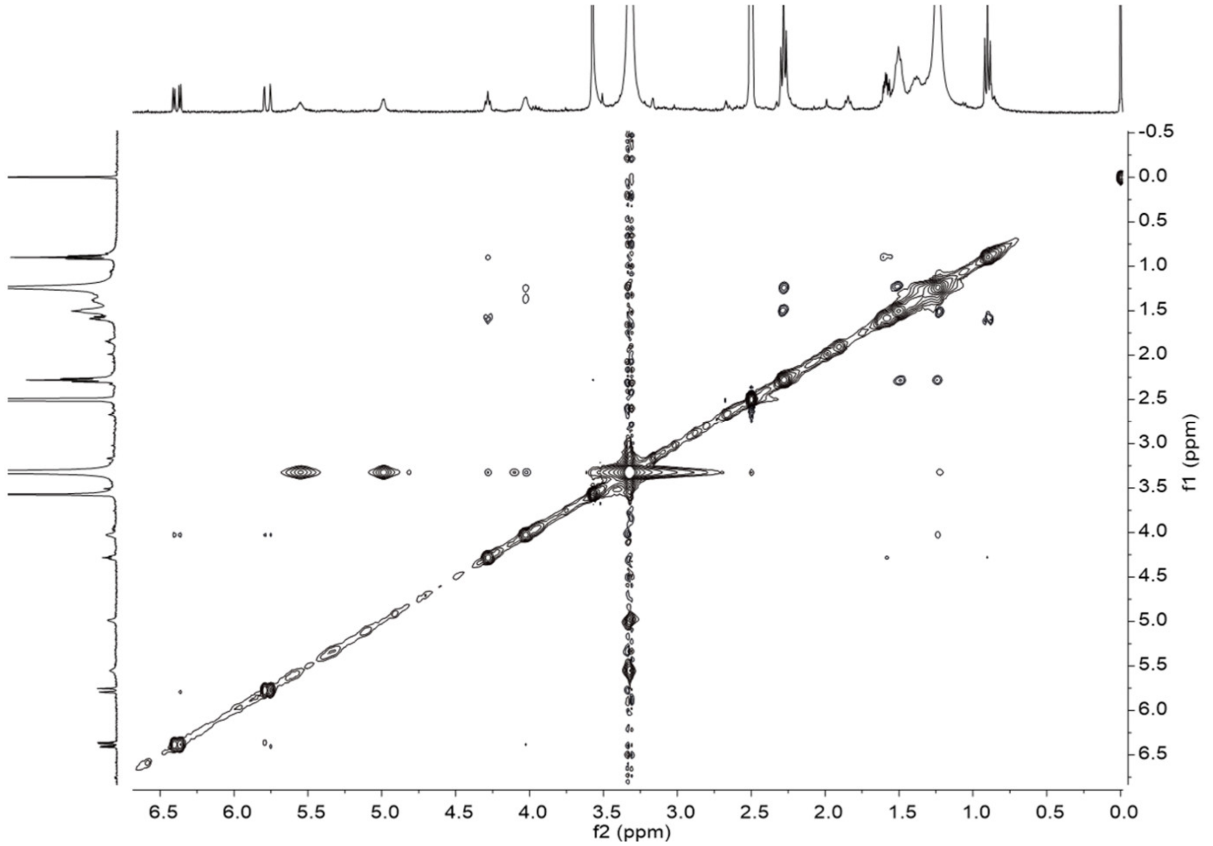

Figure S18. NOESY spectrum of **2** (400 MHz, DMSO-d_6).

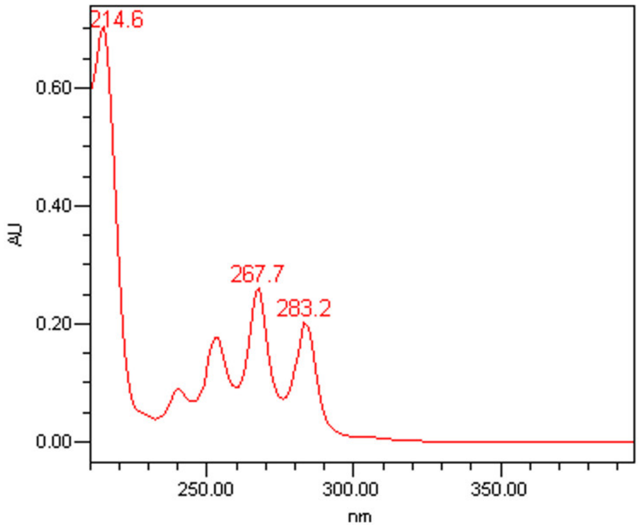

Figure S19. UV spectrum of 3.

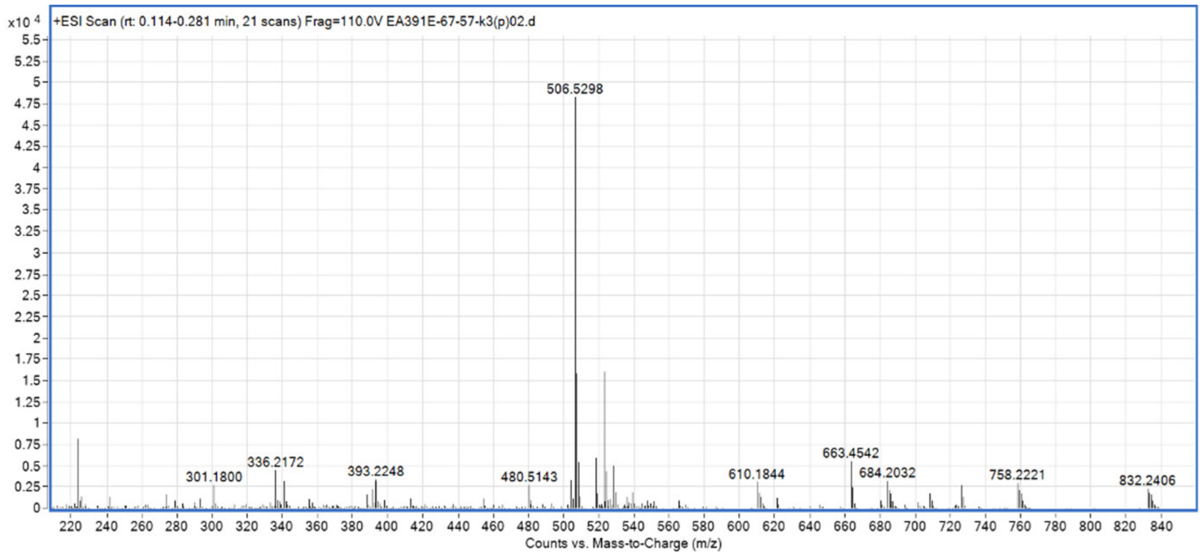

Figure S20. (+)-HR-ESI-MS spectrum of **3**.

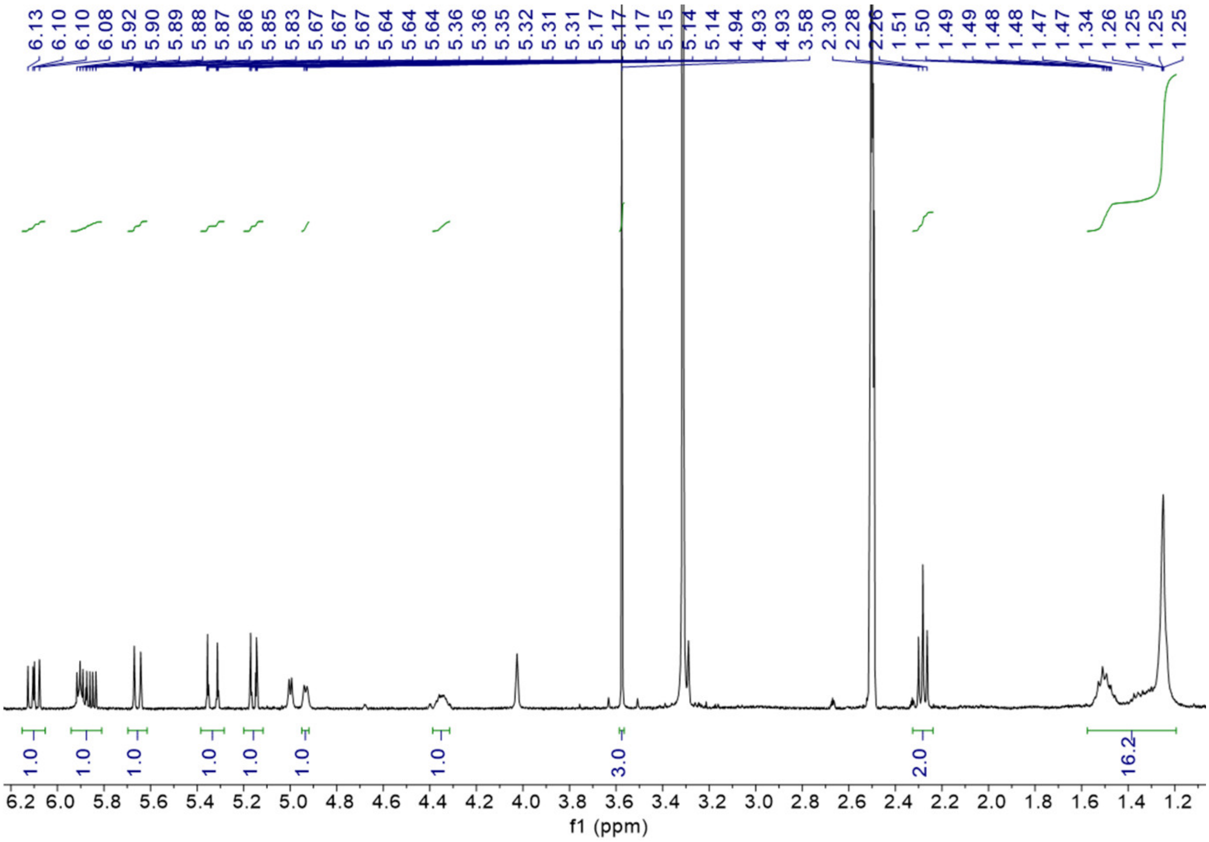

Figure S21. ^{1}H NMR spectrum of **3** (400 MHz, DMSO-d_6).

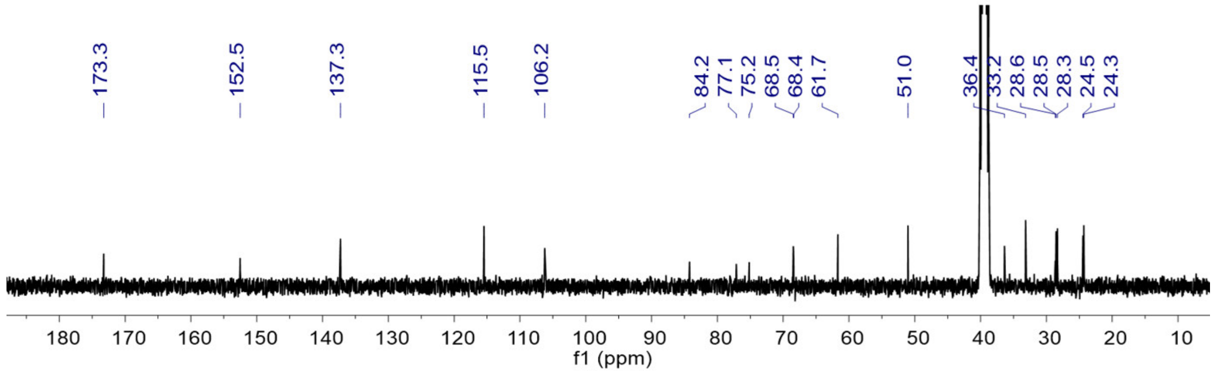

Figure S22. ^{13}C NMR spectrum of **3** (100 MHz, DMSO-d_6).

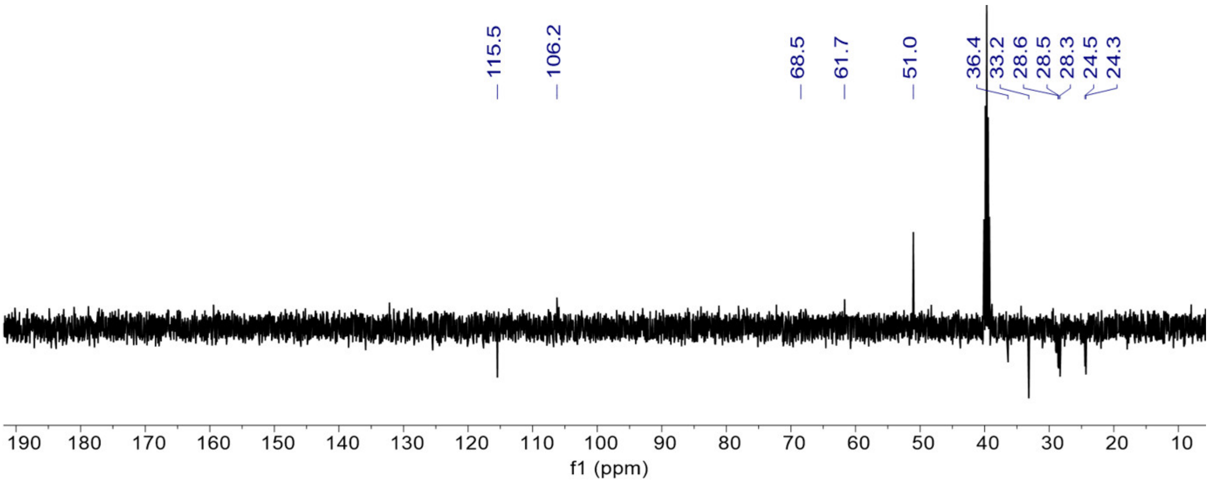

Figure S23. DEPT135 spectrum of **3** (100 MHz, DMSO-d_6).

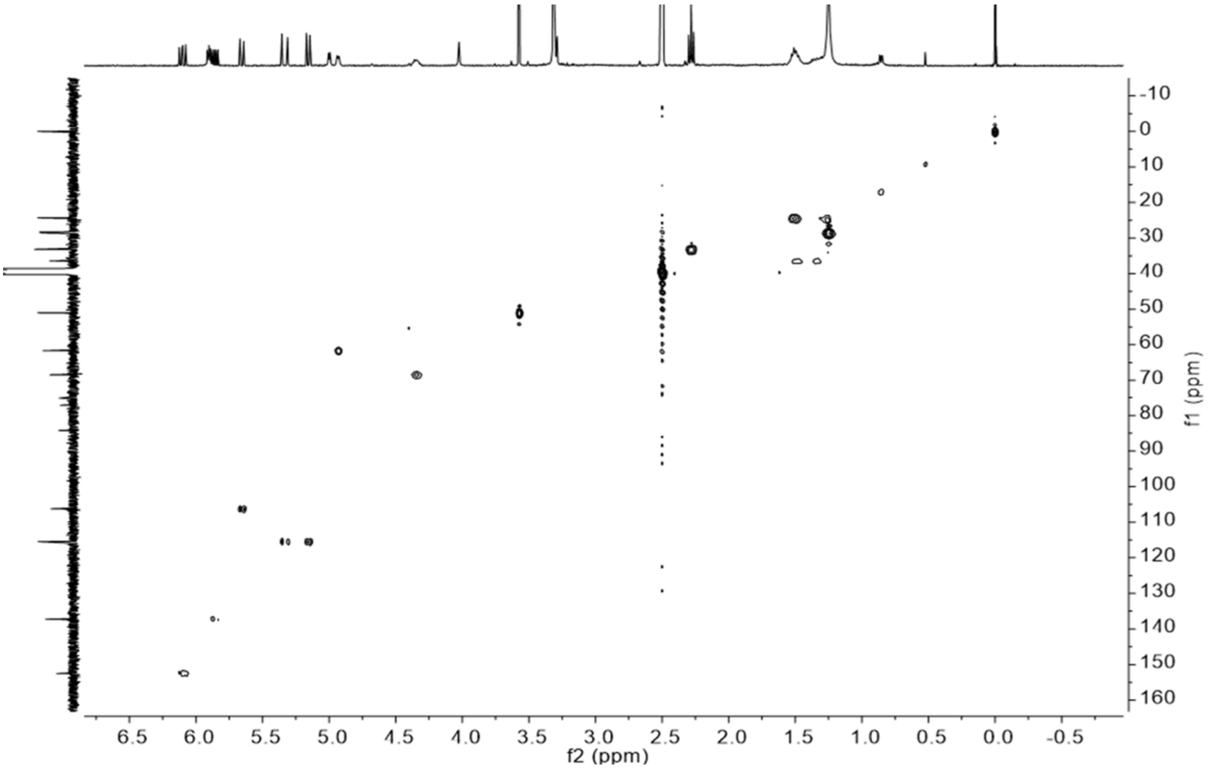

Figure S24. HSQC spectrum of **3** (^{1}H: 400 MHz, ^{13}C: 100 MHz, DMSO-d_6).

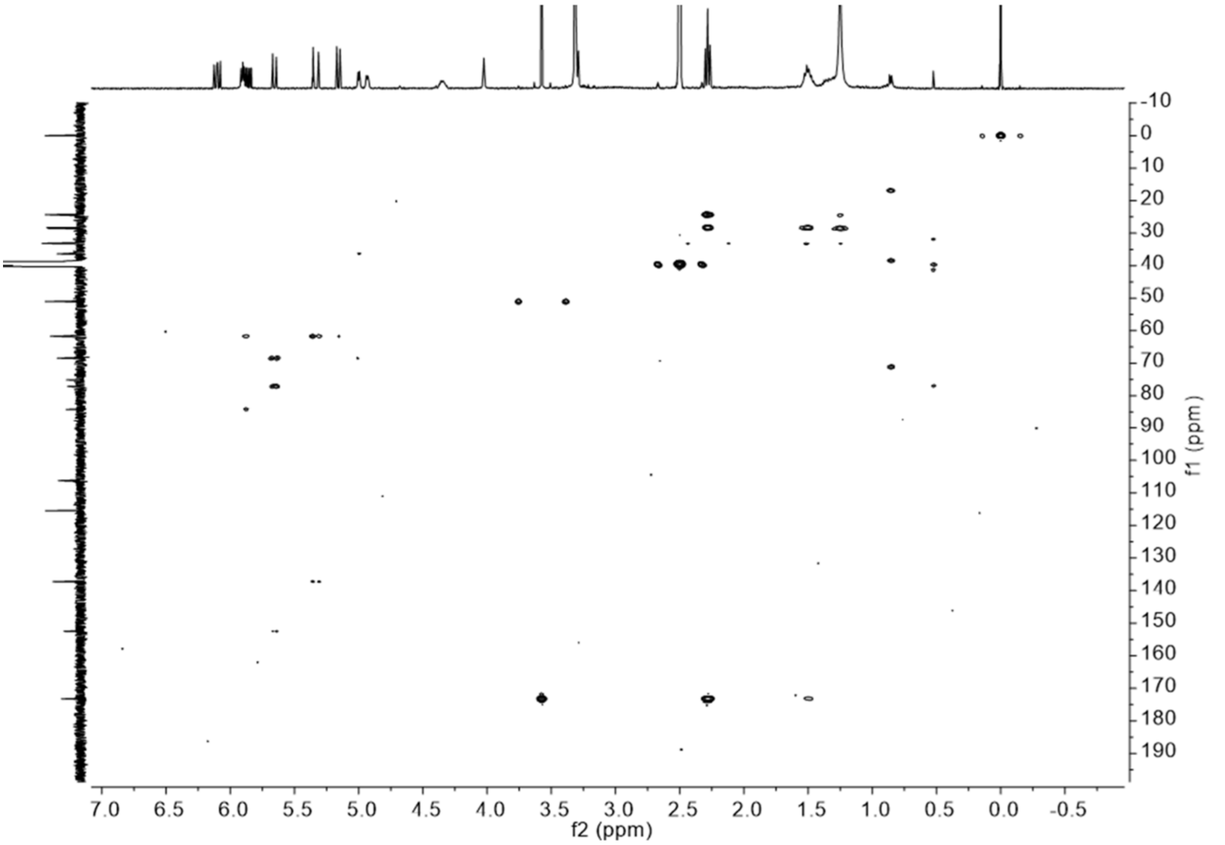

Figure S25. HMBC spectrum of **3** (^{1}H: 400 MHz, ^{13}C: 100 MHz, DMSO-d_6).

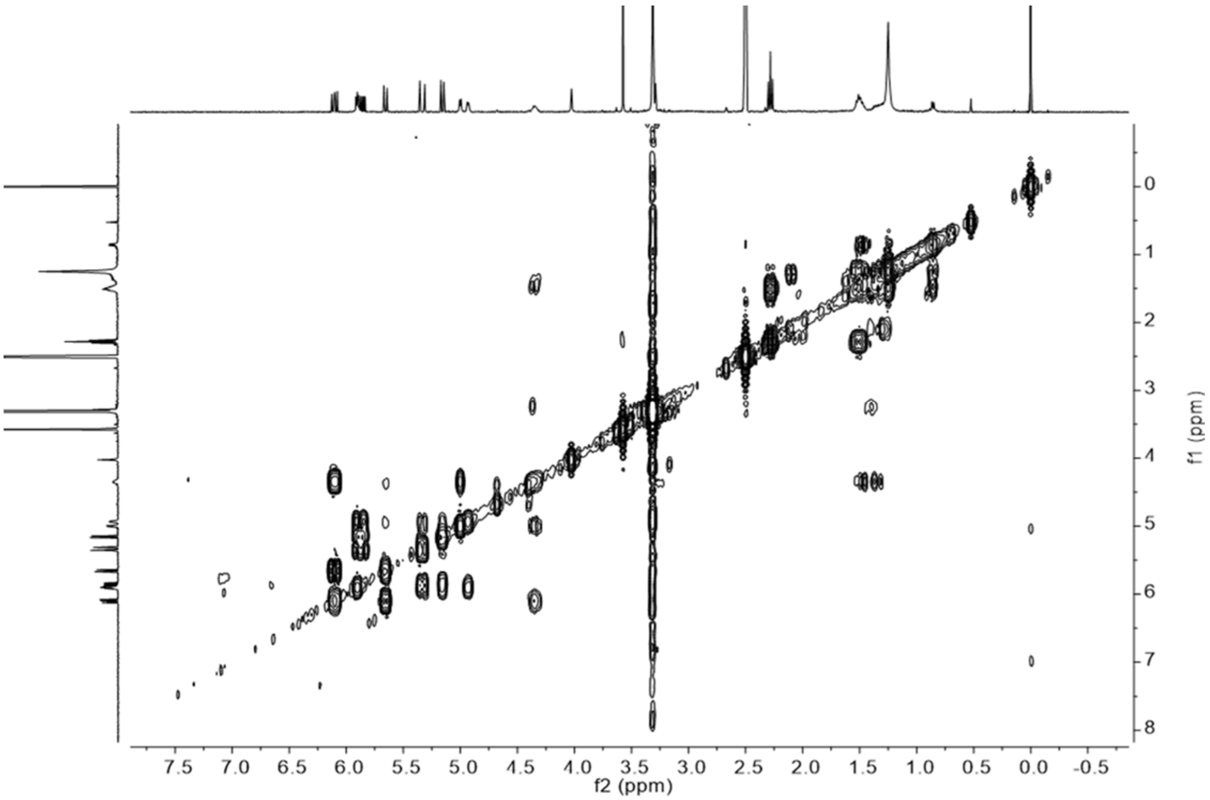

Figure S26. ^{1}H-^{1}H COSY spectrum of **3** (400 MHz, DMSO-d_6).

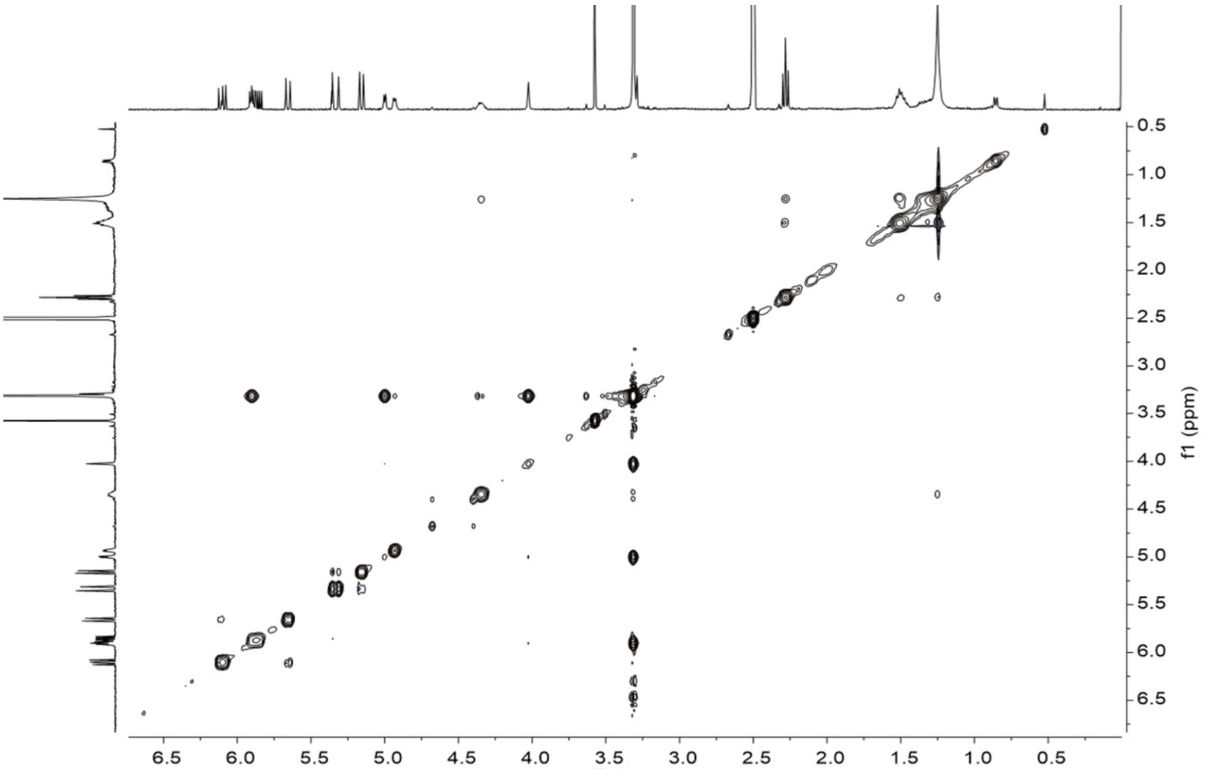

Figure S27. NOESY spectrum of **3** (400 MHz, DMSO-d_6).

9R*16S*	9R*16R*

^{13}C NMR

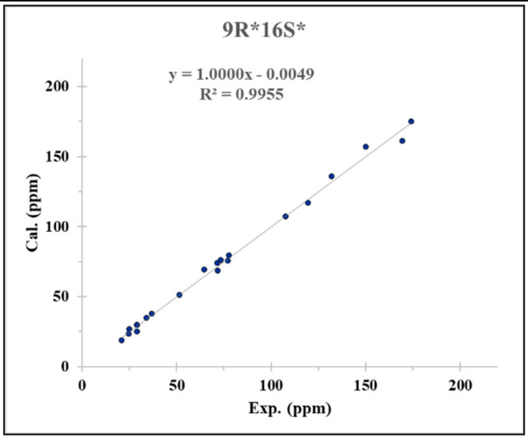

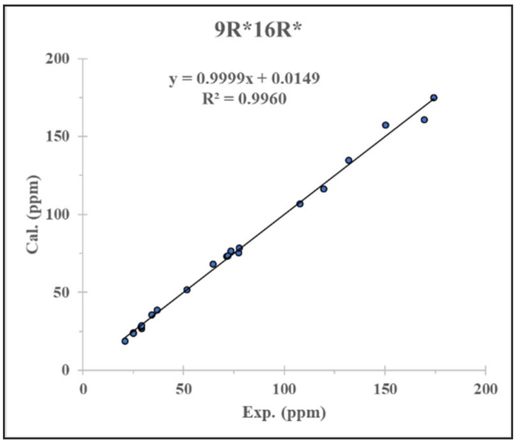

^{1}H NMR

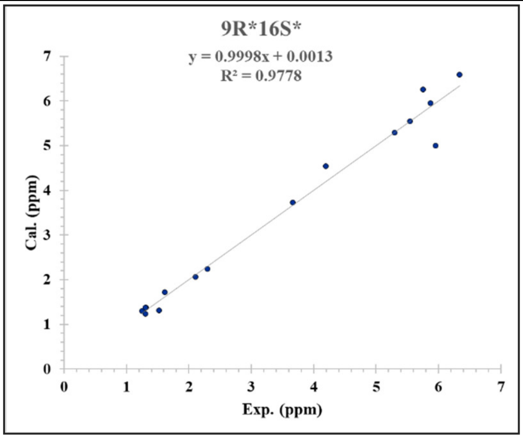

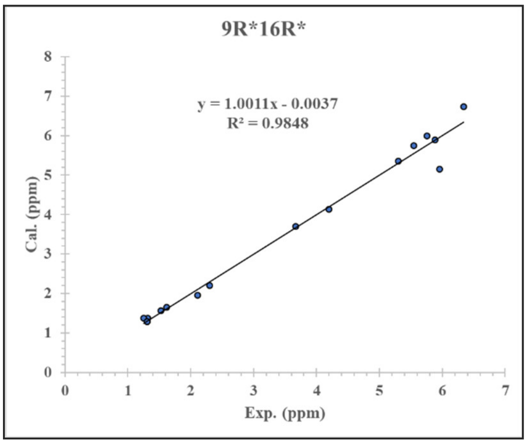

Figure S28. Linear correlation plots between calculated and experimental ^{1}H and ^{13}C NMR chemical shift values for two potential diastereomers (9*R**16*S** and 9*R**16*R**) of **1**.

Settings	Type of data (shifts)
Default	Shielding tensors

Functional	Solvent?	Basis Set
B3LYP	Gas Phase	6-311G(d,p)

TMS 1H	31.915	TMS 13C	183.890
Default	μ	σ	ν
13Cu,sp2	6.538	2.241	5.026
13Cu,sp3	5.265	2.212	14.147
1Hu,sp2	0.112	0.149	7.274
1Hu,sp3	-0.072	0.112	3.342
13Cs	-	1.837	6.341
1Hs	-	0.113	3.197

Isomer Nº			1	2	3	4	5	6	7	8
DP4+ (%)		H data	3.46%	96.54%	-	-	-	-	-	-
		C data	2.42%	97.58%	-	-	-	-	-	-
		All data	0.09%	99.91%	-	-	-	-	-	-
Type	sp2?	Exp	1	2	3	4	5	6	7	8
C	X	119.6	60.40735641	60.8340886						
C	X	132.1	41.0095887	41.8870708						
C		64.7	109.6897439	110.91252						
C	X	77.2	102.9671251	103.19368						
C	X	71.5	104.7445734	105.563411						
C	X	73.5	102.6175257	102.169833						
C	X	77.7	99.17539216	100.32206						
C	X	107.9	70.57401225	70.6230581						
C	X	150.2	18.9454725	18.2633547						
C		72	110.6705974	105.159429						
C		36.8	142.0282944	141.547034						
C		25.1	153.6439763	156.698553						
C		29.2	155.3210174	153.55097						
C		29	150.4839323	152.721675						
C		29.1	150.3461277	151.854059						
C		24.9	157.1868796	156.819054						
C		34.1	145.3037731	144.684953						
C	X	174.3	0.174867677	-0.12217514						
C	X	169.5	14.87245079	14.9098894						
C		20.9	161.650614	162.228722						
C		51.5	128.0643404	128.084079						
H	X	5.3	26.32310704	26.2777883						
H	X	5.55	26.07697948	25.8811495						
H	X	5.88	25.67128166	25.7476671						
H		5.96	26.62017577	26.4801907						
H	X	5.76	25.36821915	25.6374017						
H	X	6.34	25.03374851	24.9029087						
H		4.2	27.08865805	27.4895159						
H		1.53	30.30186001	30.0310118						
H		1.32	30.24793124	30.2183316						
H		1.25	30.31423149	30.2081945						
H		1.31	30.37966467	30.2985728						
H		1.31	30.24642769	30.307001						
H		1.62	29.89922048	29.940534						
H		2.3	29.38647063	29.3917329						
H		2.11	29.55793262	29.6492777						
H		3.67	27.89833757	27.9123006						

Figure S29. DP4+ probability for diastereomer 1 (9*R**16*S**) and diastereomer 2 (9*R**16*R**) of **1**.

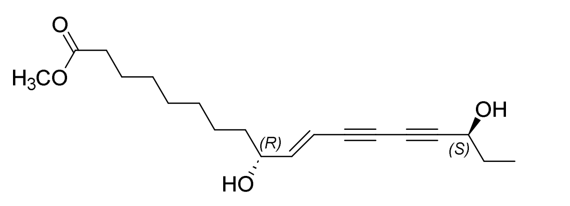

9*R**16*S**

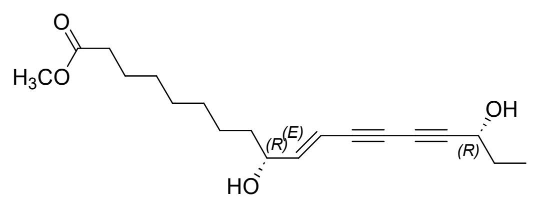

9*R**16*R**

^{13}C NMR

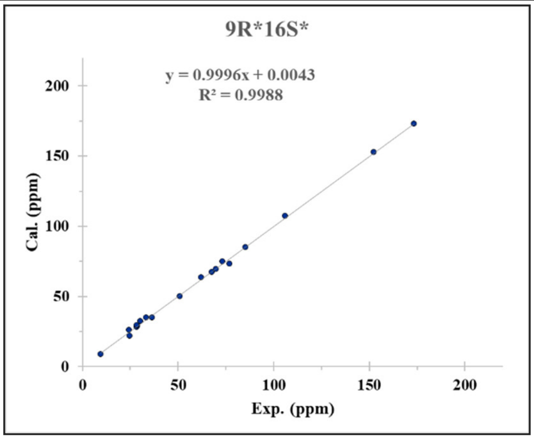

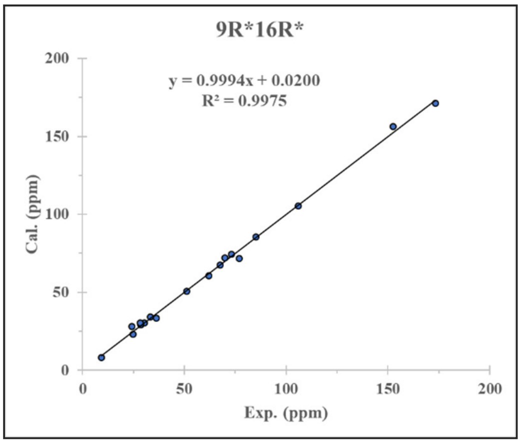

^{1}H NMR

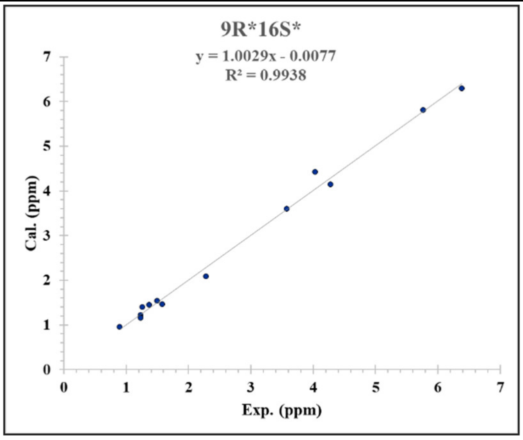

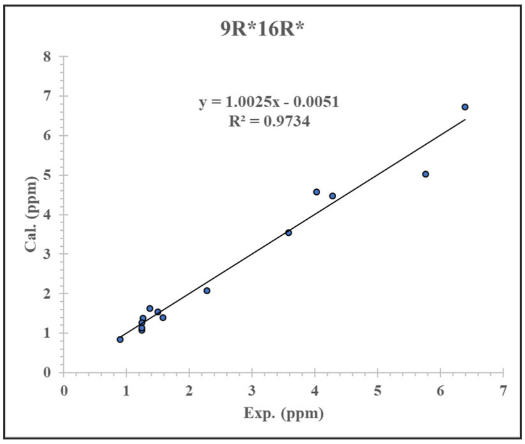

Figure S30. Linear correlation plots between calculated and experimental ^{1}H and ^{13}C NMR chemical shift values for two potential diastereomers (9*R**16*S** and 9*R**16*R**) of **2**.

Settings	Type of data (shifts)
Default	Shielding tensors

Functional	Solvent?	Basis Set
B3LYP	Gas Phase	6-311G(d,p)

TMS 1H	31.915	TMS 13C	183.890
Default	μ	σ	ν
13Cu,sp2	6.538	2.241	5.026
13Cu,sp3	5.265	2.212	14.147
1Hu,sp2	0.112	0.149	7.274
1Hu,sp3	-0.072	0.112	3.342
13Cs	-	1.837	6.341
1Hs	-	0.113	3.197

Isomer Nº			1	2	3	4	5	6	7	8
DP4+ (%)		H data	100.00%	0.00%	-	-	-	-	-	-
		C data	100.00%	0.00%	-	-	-	-	-	-
		All data	100.00%	0.00%	-	-	-	-	-	-
Type	sp2?	Exp	1	2	3	4	5	6	7	8
C		9.4	169.3408608	169.628714						
C		30.3	145.3579473	146.816309						
C		62	113.2150575	115.350637						
C	X	85.3	91.58729059	89.6828276						
C	X	67.6	109.6445659	108.453139						
C	X	73.1	101.747792	101.344581						
C	X	76.9	103.3635616	103.928087						
C	X	105.9	68.8164167	69.3471337						
C	X	152.5	22.24208797	16.5359472						
C		69.8	107.1446934	103.696186						
C		36.3	142.7001759	143.464936						
C		24.7	155.9494499	154.088067						
C		28.5	149.5897567	147.79713						
C		28.7	148.6787211	147.769069						
C		28.3	148.1777154	146.745569						
C		24.3	151.8164753	149.234085						
C		33.2	142.8013371	142.561239						
C	X	173.3	1.419351024	1.28986139						
C		51.1	127.2091701	125.825254						
H		0.9	30.58314201	30.6279191						
H		1.58	30.07019634	30.0605759						
H		4.28	27.33845357	26.9590081						
H	X	5.77	25.63968122	26.4082777						
H	X	6.39	25.15858484	24.7001071						
H		4.03	27.05566846	26.8531276						
H		1.37	30.07427141	29.8352099						
H		1.26	30.1389001	30.0875745						
H		1.24	30.30720937	30.1901857						
H		1.24	30.35869502	30.3856426						
H		1.24	30.38413959	30.3332699						
H		1.5	29.99176963	29.9234512						
H		2.28	29.43847952	29.3902695						
H		3.58	27.88758437	27.903679						

Figure S31. DP4+ probability for diastereomer 1 (9*R**16*S**) and diastereomer 2 (9*R**16*R**) of **2**.

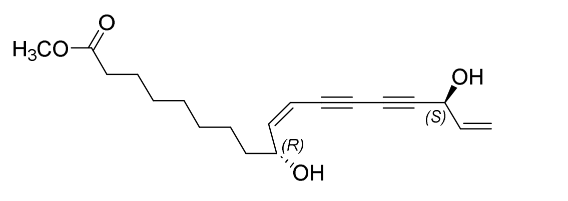

9*R**16*S**

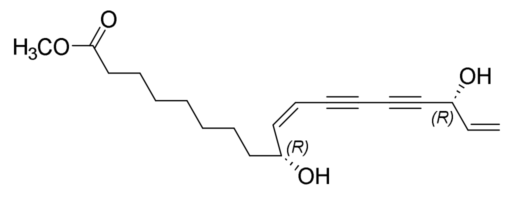

9*R**16*R**

^{13}C NMR

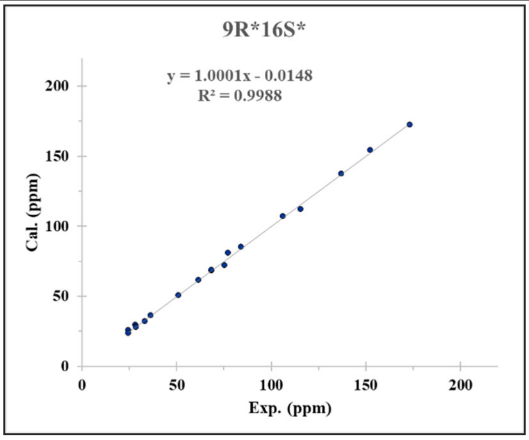

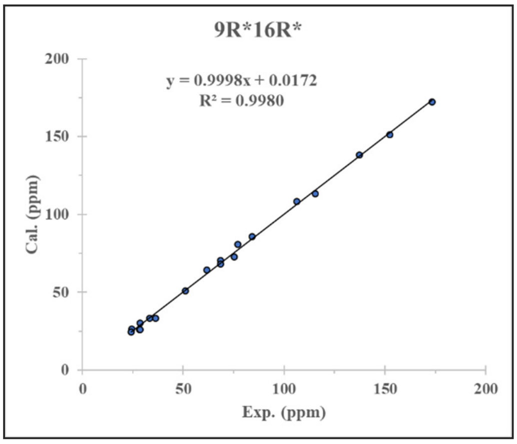

^{1}H NMR

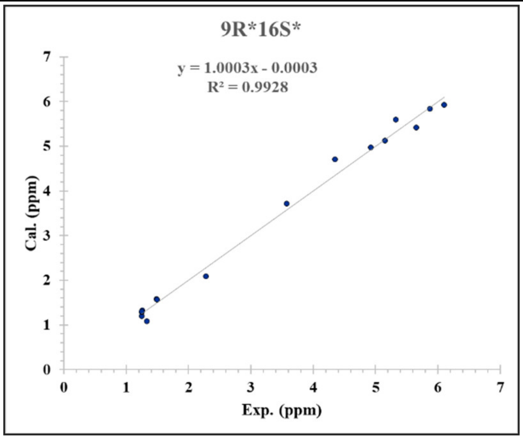

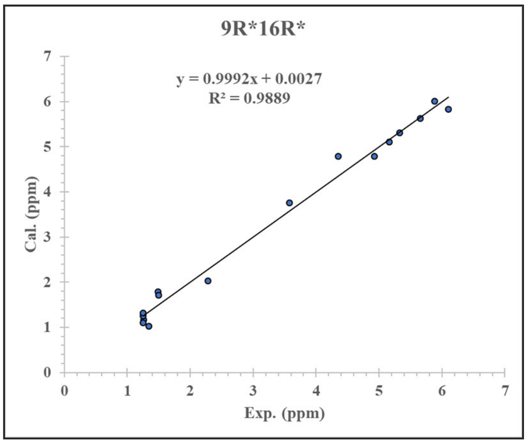

Figure S32. Linear correlation plots between calculated and experimental ^{1}H and ^{13}C NMR chemical shift values for two potential diastereomers (9*R**16*S** and 9*R**16*R**) of **3**.

Settings	Type of data (shifts)
Default	Shielding tensors

Functional	Solvent?	Basis Set
B3LYP	Gas Phase	6-311G(d,p)

TMS 1H	31.915	TMS 13C	183.890
Default	μ	σ	ν
13Cu,sp2	6.538	2.241	5.026
13Cu,sp3	5.265	2.212	14.147
1Hu,sp2	0.112	0.149	7.274
1Hu,sp3	-0.072	0.112	3.342
13Cs	-	1.837	6.341
1Hs	-	0.113	3.197

Isomer Nº			1	2	3	4	5	6	7	8
DP4+ (%)		H data	99.66%	0.34%	-	-	-	-	-	-
		C data	100.00%	0.00%	-	-	-	-	-	-
		All data	100.00%	0.00%	-	-	-	-	-	-
Type	sp2?	Exp	1	2	3	4	5	6	7	8
C	X	115.5	64.66453364	63.5184095						
C	X	137.3	38.30608104	37.1386917						
C		61.7	117.2790163	115.233555						
C	X	84.2	92.59119869	92.7016013						
C	X	68.4	110.127806	108.580416						
C	X	77.1	97.23889933	97.9082771						
C	X	75.2	106.289237	106.526197						
C	X	106.2	70.08285586	68.8550144						
C	X	152.5	21.22940238	23.3305411						
C		68.5	109.6967075	111.381305						
C		36.4	143.6666679	148.105659						
C		24.5	154.5881005	155.123095						
C		28.3	150.636181	155.644637						
C		28.5	152.0118825	155.80378						
C		28.6	152.300007	151.351171						
C		24.3	156.9382112	156.995297						
C		33.2	147.9271589	147.737808						
C	X	173.3	2.212584359	1.14280205						
C		51.0	128.7985531	129.202585						
H	X	5.16	26.46955757	26.5353081						
H	X	5.33	25.99762034	26.3307242						
H	X	5.88	25.74684862	25.6435438						
H		4.93	26.62355758	26.8395982						
H	X	5.66	26.17036416	26.0178552						
H	X	6.10	25.66332802	25.8224296						
H		4.35	26.8936592	26.8422917						
H		1.34	30.55361244	30.5223952						
H		1.49	30.05851755	29.7782865						
H		1.26	30.31848865	30.3959167						
H		1.25	30.44670569	30.302032						
H		1.25	30.35571597	30.4491602						
H		1.25	30.34252374	30.2378987						
H		1.5	30.07527915	29.8494592						
H		2.28	29.54362077	29.5389435						
H		3.58	27.90008456	27.8513964						

Figure S33. DP4+ probability for diastereomer 1 (9*R**16*S**) and diastereomer 2 (9*R**16*R**) of **3**.

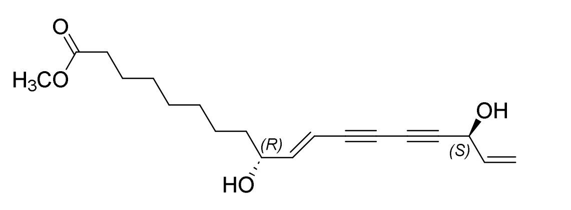

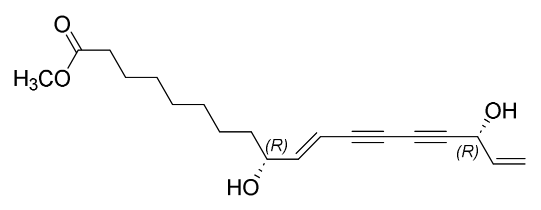

9*R**16*S**	9*R**16*R**

^{13}C NMR

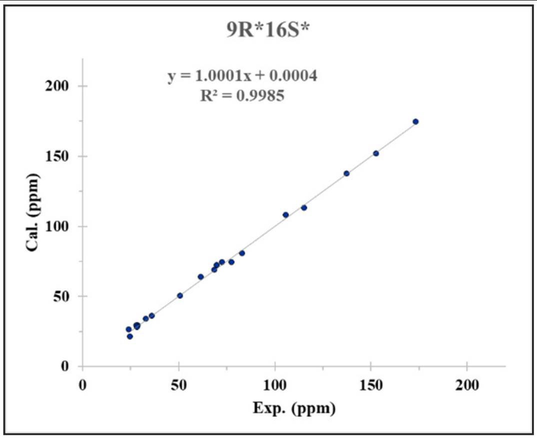

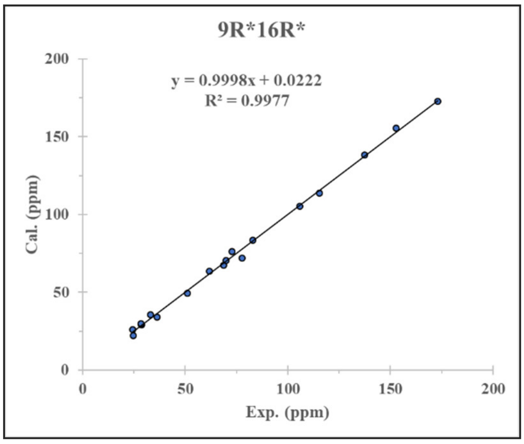

^{1}H NMR

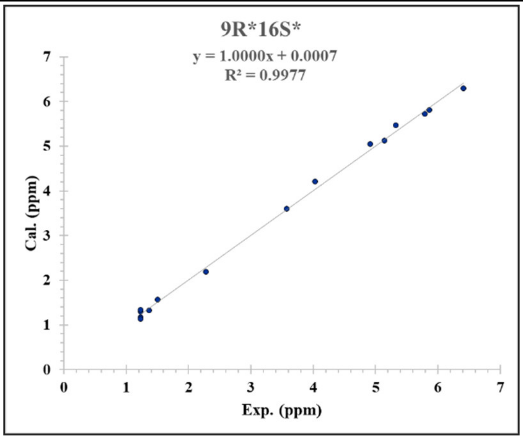

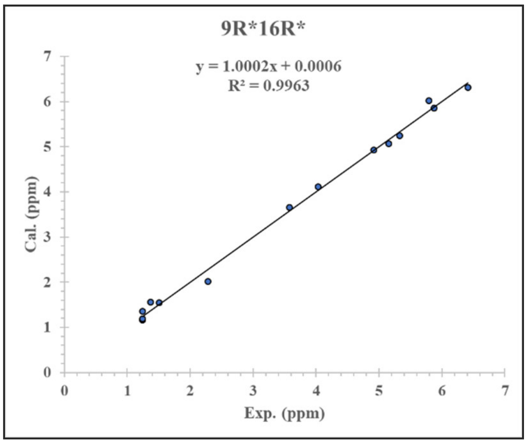

Figure S34. Linear correlation plots between calculated and experimental ^{1}H and ^{13}C NMR chemical shift values for two potential diastereomers (9*R**16*S** and 9*R**16*R**) of **4**.

Settings	Type of data (shifts)
Default	Shielding tensors

Functional	Solvent?	Basis Set
B3LYP	Gas Phase	6-311G(d,p)

TMS 1H	31.915	TMS 13C	183.890
Default	μ	σ	ν
13Cu,sp2	6.538	2.241	5.026
13Cu,sp3	5.265	2.212	14.147
1Hu,sp2	0.112	0.149	7.274
1Hu,sp3	-0.072	0.112	3.342
13Cs	-	1.837	6.341
1Hs	-	0.113	3.197

Isomer №		1	2	3	4	5	6	7	8
DP4+ (%)	H data	93.37%	6.63%	-	-	-	-	-	-
	C data	100.00%	0.00%	-	-	-	-	-	-
	All data	100.00%	0.00%	-	-	-	-	-	-

Type	sp2?	Exp	1	2	3	4	5	6	7	8
C	X	115.4	63.0727555	62.0937866						
C	X	137.4	38.20867096	36.8814977						
C		61.7	113.5540274	112.674157						
C	X	83	96.28118968	92.8127136						
C	X	68.7	108.2259834	109.172132						
C	X	72.8	102.5123218	100.168261						
C	X	77.6	102.4708148	104.128024						
C	X	105.8	68.11206239	70.5440278						
C	X	152.8	23.77705199	19.7074765						
C		69.8	105.0654373	105.79904						
C		36.3	141.6058308	142.855366						
C		24.7	156.604851	154.854374						
C		28.7	148.5374695	147.767194						
C		28.3	148.7524689	147.626046						
C		28.5	149.9761336	147.027732						
C		24.3	151.7628928	150.886793						
C		33.2	143.7429776	141.219735						
C	X	173.2	0.54558777	2.21788855						
C		51	127.1892234	127.355903						
H	X	5.15	26.40777304	26.4592952						
H	X	5.33	26.06966212	26.2856428						
H	X	5.87	25.72863698	25.6798017						
H		4.92	26.48828746	26.5986293						
H	X	5.79	25.81170018	25.5200913						
H	X	6.41	25.23649189	25.2284532						
H		4.03	27.31684359	27.420751						
H		1.37	30.21936011	29.9522553						
H		1.24	30.24130841	30.1637828						
H		1.24	30.2106011	30.3371074						
H		1.24	30.36646325	30.3407667						
H		1.24	30.40549833	30.3249559						
H		1.51	29.97960997	29.9674281						
H		2.28	29.35379657	29.4999075						
H		3.58	27.9415408	27.8624621						

Figure S35. DP4+ probability for diastereomer 1 (9*R**16*S**) and diastereomer 2 (9*R**16*R**) of **4**.

Table S1. Energy analysis for diastereomer 1 (9*R**16*S**) and diastereomer 2 (9*R**16*R**) of **1**.

Conformer	DFT energy (Hartree)	ΔE（Kcal/mol）	Boltzmann distribution (%)
		9*R**16*S**	
1	-1191.426082	0.00	46.36
2	-1191.425967	0.07	41.05
3	-1191.424594	0.93	9.59
4	-1191.422858	2.02	1.52
5	-1191.422828	2.04	1.48
		9*R**16*R**	
1	-1191.423072	0.00	39.94
2	-1191.422684	0.24	26.50
3	-1191.421218	1.16	5.61
4	-1191.421154	1.20	5.24
5	-1191.421126	1.22	5.09
6	-1191.421059	1.26	4.74
7	-1191.421045	1.27	4.67
8	-1191.420348	1.71	2.23
9	-1191.420264	1.76	2.04
10	-1191.420108	1.86	1.73
11	-1191.419704	2.11	1.13
12	-1191.419676	2.13	1.09

Table S2. Calculated (calcd.) and experimental (exp.) ^{13}C chemical shift values for diastereomer 1 (9*R**16*S**) and diastereomer 2 (9*R**16*R**) of **1**.

Exp.	Calc. for 1	|Δδ|	Calc. for 2	|Δδ|
119.6	116.8	2.8	116.3	3.3
132.1	135.6	3.5	134.6	2.5
64.7	69.1	4.4	68.0	3.3
77.2	75.6	1.6	75.5	1.7
71.5	73.9	2.4	73.2	1.7
73.5	75.9	2.4	76.5	3.0
77.7	79.3	1.6	78.3	0.6
107.9	107.0	0.9	106.9	1.0
150.2	157.0	6.8	157.4	7.2
72	68.2	3.8	73.6	1.6
36.8	37.8	1.0	38.5	1.7
25.1	26.5	1.4	23.9	1.2
29.2	24.9	4.3	26.9	2.3
29	29.6	0.6	27.7	1.3
29.1	29.7	0.6	28.6	0.5
24.9	23.1	1.8	23.8	1.1
34.1	34.6	0.5	35.5	1.4
174.3	175.1	0.8	175.1	0.8
169.5	160.9	8.6	160.6	8.9
20.9	18.8	2.1	18.6	2.3
51.5	51.3	0.2	51.5	0.0

Table S3. Calculated (calcd.) and experimental (exp.) ^{1}H chemical shift values for diastereomer 1 (9*R**16*S**) and diastereomer 2 (9*R**16*R**) of **1**.

Exp.	Calc. for 1	|Δδ|	Calc. for 2	|Δδ|
5.30	5.29	0.01	5.35	0.1
5.55	5.54	0.01	5.75	0.20
5.88	5.94	0.06	5.89	0.01
5.96	4.99	0.97	5.15	0.81
5.76	6.25	0.49	6.00	0.24

6.34	6.58	0.24	6.74	0.40
4.20	4.53	0.33	4.13	0.07
1.53	1.31	0.22	1.56	0.03
1.32	1.37	0.05	1.37	0.05
1.25	1.30	0.05	1.38	0.13
1.31	1.23	0.08	1.29	0.02
1.31	1.37	0.06	1.29	0.02
1.62	1.71	0.09	1.65	0.03
2.30	2.23	0.07	2.21	0.09
2.11	2.06	0.05	1.95	0.16
3.67	3.72	0.05	3.70	0.03

Table S4. Energy analysis for diastereomer 1 (9*R**16*S**) and diastereomer 2 (9*R**16*R**) of **2**.

Conformer	DFT energy (Hartree)	ΔE（Kcal/mol）	Boltzmann distribution (%)
	9*R**16*S**		
1	-1040.192491	0.00	31.11
2	-1040.19236	0.08	27.09
3	-1040.19222	0.17	23.35
4	-1040.191543	0.59	11.40
5	-1040.190303	1.37	3.07
6	-1040.189821	1.68	1.84
7	-1040.189313	1.99	1.07
8	-1040.189295	2.01	1.05
	9*R**16*R**		
1	-1040.191475	0.00	41.23
2	-1040.191448	0.02	40.08
3	-1040.189597	1.18	5.64
4	-1040.189361	1.33	4.39
5	-1040.189243	1.40	3.88
6	-1040.189203	1.43	3.72
7	-1040.188015	2.17	1.06

Table S5. Calculated (calc.) and experimental (exp.) ^{13}C chemical shift values for diastereomer 1 (9*R**16*S**) and diastereomer 2 (9*R**16*R**) of **2**.

Exp.	Calc. for 1	|Δδ|	Calc. for 2	|Δδ|
9.4	8.7	0.7	8.1	1.3
30.3	32.2	1.9	30.2	0.1
62	63.7	1.7	60.7	1.3
85.3	84.8	0.5	85.5	0.2
67.6	67.1	0.5	67.4	0.2
73.1	74.9	1.8	74.3	1.2
76.9	73.3	3.6	71.7	5.2
105.9	107.1	1.2	105.3	0.6
152.5	152.7	0.2	156.4	3.9
69.8	69.6	0.2	72.0	2.2
36.3	34.8	1.5	33.4	2.9
24.7	21.8	2.9	23.2	1.5
28.5	28.1	0.4	29.2	0.7
28.7	29.0	0.3	29.3	0.6
28.3	29.4	1.1	30.3	2.0
24.3	25.9	1.6	27.9	3.6
33.2	34.7	1.5	34.3	1.1
173.3	173.0	0.3	171.2	2.1
51.1	50.0	1.1	50.5	0.6

Table S6. Calculated (calc.) and experimental (exp.) ^{1}H chemical shift values for diastereomer 1 (9*R**16*S**) and diastereomer 2 (9*R**16*R**) of **2**.

Exp.	Calc. for 1	|Δδ|	Calc. for 2	|Δδ|
0.9	0.95	0.05	0.84	0.06
1.58	1.46	0.12	1.40	0.18
4.28	4.14	0.14	4.48	0.20
5.77	5.81	0.04	5.02	0.75
6.39	6.29	0.10	6.72	0.33
4.03	4.42	0.39	4.58	0.55
1.37	1.45	0.08	1.63	0.26
1.26	1.39	0.13	1.38	0.12
1.24	1.22	0.02	1.27	0.03
1.24	1.17	0.07	1.08	0.16
1.24	1.15	0.09	1.13	0.11
1.50	1.53	0.03	1.54	0.04
2.28	2.08	0.20	2.07	0.21
3.58	3.60	0.02	3.54	0.04

Table S7. Energy analysis for diastereomer 1 (9*R**16*S**) and diastereomer 2 (9*R**16*R**) of **3**.

Conformer	DFT energy (Hartree)	ΔE（Kcal/mol）	Boltzmann distribution (%)
		9*R**16*S**	
1	-1039.182497	0.00	44.33
2	-1039.181819	0.43	21.62
3	-1039.180945	0.97	8.57
4	-1039.180646	1.16	6.24
5	-1039.180179	1.45	3.81
6	-1039.180154	1.47	3.71
7	-1039.180152	1.47	3.70
8	-1039.179994	1.57	3.13
9	-1039.179773	1.71	2.48
10	-1039.179754	1.72	2.43
		9*R**16*R**	
1	-1039.183734	0.00	70.32
2	-1039.182765	0.61	25.19
3	-1039.180514	2.02	2.32
4	-1039.180451	2.06	2.17

Table S8. Calculated (calc.) and experimental (exp.) ^{13}C chemical shift values for diastereomer 1 (9*R**16*S**) and diastereomer 2 (9*R**16*R**) of **3**.

Exp.	Calc. for 1	|Δδ|	Calc. for 2	|Δδ|
115.5	112.3	3.2	113.2	2.3
137.3	137.6	0.3	138.2	0.9
61.7	61.6	0.1	64.2	2.5
84.2	85.4	1.2	85.6	1.4
68.4	68.5	0.1	70.5	2.1
77.1	80.9	3.8	80.6	3.5
75.2	72.2	3.0	72.5	2.7
106.2	107.1	0.9	108.2	2.0
152.5	154.1	1.6	151.3	1.2
68.5	68.9	0.4	67.9	0.6
36.4	36.2	0.2	33.1	3.3
24.5	25.7	1.2	26.4	1.9
28.3	29.5	1.2	25.9	2.4
28.5	28.2	0.3	25.8	2.7
28.6	27.9	0.7	30.0	1.4

24.3	23.5	0.8	24.6	0.3
33.2	32.1	1.1	33.4	0.2
173.3	172.4	0.9	172.3	1.0
51.0	50.5	0.5	51.0	0.0

Table S9. Calculated (calc.) and experimental (exp.) ^{1}H chemical shift values for diastereomer 1 (9*R**16*S**) and diastereomer 2 (9*R**16*R**) of **3**.

**Exp.**	**Calc. for 1**	**|Δδ|**	**Calc. for 2**	**|Δδ|**
5.16	5.12	0.04	5.10	0.06
5.33	5.59	0.26	5.31	0.02
5.88	5.83	0.05	6.01	0.13
4.93	4.97	0.04	4.79	0.14
5.66	5.41	0.25	5.63	0.03
6.10	5.92	0.18	5.83	0.27
4.35	4.70	0.35	4.79	0.44
1.34	1.08	0.26	1.03	0.31
1.49	1.57	0.08	1.79	0.30
1.26	1.32	0.06	1.16	0.10
1.25	1.19	0.06	1.25	0.00
1.25	1.28	0.03	1.10	0.15
1.25	1.29	0.04	1.32	0.07
1.50	1.56	0.06	1.71	0.21
2.28	2.08	0.20	2.03	0.25
3.58	3.71	0.13	3.76	0.18

Table S10. Energy analysis for diastereomer 1 (9*R**16*S**) and diastereomer 2 (9*R**16*R**) of **4**.

**Conformer**	**DFT energy (Hartree)**	**ΔE（Kcal/mol）**	**Boltzmann distribution (%)**
		9*R**16*S**	
1	-1038.964879	0.00	53.11
2	-1038.963292	1.00	9.89
3	-1038.963009	1.17	7.33
4	-1038.962970	1.20	7.03
5	-1038.962819	1.29	5.99
6	-1038.962724	1.35	5.42
7	-1038.962578	1.44	4.64
8	-1038.962364	1.58	3.70
9	-1038.961498	2.12	1.48
10	-1038.961450	2.15	1.41
		9*R**16*R**	
1	-1038.966095	0.00	66.99
2	-1038.963873	1.39	6.37
3	-1038.963807	1.44	5.94
4	-1038.963581	1.58	4.67
5	-1038.963531	1.61	4.43
6	-1038.963498	1.63	4.28
7	-1038.963102	1.88	2.82
8	-1038.962955	1.97	2.41
9	-1038.962821	2.05	2.09

Table S11. Calculated (calc.) and experimental (exp.) ^{13}C chemical shift values for diastereomer 1 (9*R**16*S**) and diastereomer 2 (9*R**16*R**) of **4**.

Exp.	Calc. for 1	|Δδ|	Calc. for 2	|Δδ|
115.4	113.2	2.2	113.6	1.8
137.4	137.6	0.2	138.4	1.0
61.7	63.6	1.9	63.7	2.0
83.0	80.6	2.4	83.3	0.3
68.7	68.8	0.1	67.2	1.5
72.8	74.5	1.7	76.0	3.2
77.6	74.5	3.1	72.1	5.5
105.8	108.2	2.4	105.2	0.6
152.8	151.7	1.1	155.3	2.5
69.8	72.0	2.2	70.5	0.7
36.3	36.1	0.2	34.0	2.3
24.7	21.4	3.3	22.2	2.5
28.7	29.3	0.6	29.1	0.4
28.3	29.1	0.8	29.3	1.0
28.5	27.9	0.6	29.9	1.4
24.3	26.1	1.8	26.1	1.8
33.2	34.0	0.8	35.6	2.4
173.2	174.5	1.3	172.5	0.7
51.0	50.2	0.8	49.3	1.7

Table S12. Calculated (calc.) and experimental (exp.) ^{1}H chemical shift values for diastereomer 1 (9*R**16*S**) and diastereomer 2 (9*R**16*R**) of **4**.

Exp.	Calc. for 1	|Δδ|	Calc. for 2	|Δδ|
5.15	5.12	0.03	5.07	0.08
5.33	5.46	0.13	5.25	0.08
5.87	5.80	0.07	5.86	0.01
4.92	5.04	0.12	4.93	0.01
5.79	5.72	0.07	6.02	0.23
6.41	6.29	0.12	6.31	0.10
4.03	4.21	0.18	4.11	0.08
1.37	1.32	0.05	1.56	0.19
1.24	1.29	0.05	1.35	0.11
1.24	1.33	0.09	1.17	0.07
1.24	1.17	0.07	1.17	0.07
1.24	1.13	0.11	1.19	0.05
1.51	1.56	0.05	1.55	0.04
2.28	2.18	0.10	2.02	0.26
3.58	3.59	0.01	3.66	0.08
